# Proteomics Reveals the Response Mechanism of Embryonic Bovine Lung Cells to *Mycoplasma bovis* Infection

**DOI:** 10.3390/ijms26020823

**Published:** 2025-01-19

**Authors:** Li Wang, Qing Wang, Yudong Liu, Yunxia Chen, Shijun Bao, Xiaoli Zhang, Chuan Wang

**Affiliations:** College of Veterinary Medicine, Gansu Agricultural University, Lanzhou 730070, China; 18193356613@163.com (L.W.); 17393189362@163.com (Q.W.); 13042950510@163.com (Y.L.); 15293890596@163.com (Y.C.); bsjdy@126.com (S.B.)

**Keywords:** *Mycoplasma bovis*, embryonic bovine lung cells, mitochondrial damage, proteomics, signaling pathway

## Abstract

*Mycoplasma bovis* (*M. bovis*) has caused huge economic losses to the cattle industry. The interaction between *M. bovis* and host cells is elucidated by screening and identifying the target protein of *M. bovis* adhesin on the surface of the host cell membrane. However, the response mechanism of embryonic bovine lung (EBL) cells to *M. bovis* infection is not yet fully understood. Additionally, it is necessary to further explore whether infection with *M. bovis* induces oxidative stress and mitochondrial damage in EBL cells. In this study, oxidation reaction, mitochondrial membrane potential, mitochondrial structure, and apoptosis ability of EBL cells infected with *M. bovis* were assessed at different times (12, 24, 48 h post-infection; hpi). Then, the differential proteomic analysis of *M. bovis*-infected EBL cells at 12 h and 24 h was performed with uninfected cells as the control. The results showed that *M. bovis* infection reduced the antioxidant capacity of EBL cells, increased ROS levels, and decreased mitochondrial membrane potential. The mitochondrial membrane of EBL cells was damaged, and the ridge arrangement was disordered after infection by transmission electron microscopy. With the increase in infection time, the mitochondrial matrix partially dissolved and spilled. The apoptosis rate of EBL cells increased with the increase in infection time of *M. bovis*. Furthermore, proteomic analysis identified 268 and 2061 differentially expressed proteins (DEPs) at 12 hpi and 24 hpi, respectively, compared with the uninfected cells. According to GO analysis, these DEPs were involved in the mitosis and negative regulation of cell growth. Additionally, the Kyoto Encyclopedia of Genes and Genomes (KEGG) analysis indicated the following pathways were linked to mitochondrial damage or cell growth regulation, including glycolysis/gluconeogenesis, pentose phosphate pathway, oxidative phosphorylation, AMPK, cGMP-PKG, cAMP, calcium, Wnt, Phospholipase D, apoptosis, MAPK, cell cycle, Ras, PI3K-Akt, mTOR, HIF-1. PPI results indicated that YWHAZ, PIK3CA, HSP90AB1, RAP1A, TXN, RAF1, MAPK1, PKM, PGK1, and GAPDH might be involved in mitochondrial pathway apoptosis induced by *M. bovis* infection. This study offers helpful data toward understanding the response of mitochondria of EBL cells to *M. bovis* infection.

## 1. Introduction

*Mycoplasma bovis* (*M. bovis*) is a cell wall-free, cell nucleus-free, and self-replicating smallest prokaryotic organism belonging to the genus Mycoplasma of the family Mycoplasma. *M. bovis* is strongly associated with diseases such as bovine respiratory disease, reproductive disorders, mastitis, and arthritis [1]. The incidence of calves is 50%~100%, and the fatality rate was as high as 10%~50%, causing significant economic damage to the cattle industry [2].

Adhesion is the first step for *M. bovis* to enter the host cell. *M. bovis* has no cell wall, and its adhesion to the host cell is mediated by the adhesion protein and the target protein on the host cell [3,4]. The number of *M. bovis* attached to embryonic bovine lung (EBL) cells increased steadily with the increase in *M. bovis* number until the EBL receptor saturation was reached [5]. As a major participant in the adhesion of *M. bovis* to different hosts, *M. bovis* adhesins can form tight binding with target proteins on the host cell membrane to cause local membrane fusion and mediate *M. bovis* invasion of cells [6]. *M. bovis* invasion is largely dependent on the internalization mediated by clavin and fossa protein and is associated with multiple activated kinases, cytoskeletal rearrangements, and morphological changes [7]. Clathrin-dependent endocytosis is one of the major pathways by which *M. bovis* invades synovial cells [8]. Following the successful invasion of host cells, mycoplasma modulates the host immune response by inducing inflammation [9,10], incomplete autophagy [11], apoptosis [12], and inhibiting the activity of immune cells or immune effectors [13]. This complex strategy allows mycoplasma to establish an elastic refuge in vesicles, cytoplasm, perinuclear, and nucleus, which is conducive to the long-term survival of mycoplasma.

Mitochondria are dynamic and highly plastic organelles of eukaryotes involved in energy production and fatty acid oxidation [14]. Mitochondria are the center of the immune system, which can regulate the innate immune pathway during pathogen infection. Mitochondria-mediated immune function mainly involves the regulation of reactive oxygen species production, inflammasome activation, cytokine secretion, and apoptosis of infected cells [15]. Mitochondrial damage is usually manifested by increased calcium and reactive oxygen species (ROS), decreased mitochondrial membrane potential (MMP), and morphological damage [16]. Recent research has shown that some pathogens target and regulate mitochondrial function to promote their own survival and growth. The SopB effector protein of *Salmonella typhimurium* binds to cytoplasmic tumor necrosis factor receptor-associated factor 6 (TRAF6), blocking mitochondrial recruitment of TRAF6 and inhibiting ROS-induced apoptosis [17]. *Mycobacterium tuberculosis* induces mitochondrial fusion and ATP activation synthesis to inhibit cell apoptosis [18,19]. However, the response of mitochondria of EBL cells to *M. bovis* infection remains unclear. To investigate which proteins and which signaling pathways lead to mitochondrial damage in *M. bovis* infection. In this study, we assessed oxidation reaction, MMP, mitochondrial structure, and apoptosis ability in both uninfected EBL cells and those infected with *M. bovis* at different times (12, 24, 48 h post-infection; hpi). Additionally, a differential proteomic analysis was conducted comparing *M. bovis*-infected cells at 12 and 24 h with uninfected controls. This study provides new data on the mechanism of the response of mitochondria of EBL cells to *M. bovis* infection.

## 2. Results

### 2.1. The Survival Rate of EBL Cells Decreased After Infection with Mycoplasma bovis

**Figure 1:**
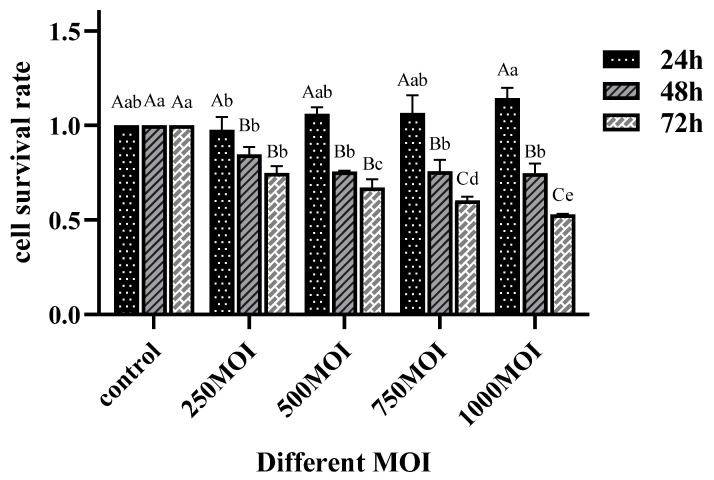
The survival rate in EBL cells were infected with different MOI of *M. bovis* at different times (24 h, 48 h, 72 h). Data are presented as mean ± standard deviation (*n* = 3). The figure contains different lowercase letters, indicating significant differences under different concentration treatment levels at the same time (*p* < 0.05). The figure contains different uppercase letters indicating significant differences at different times under the same concentration treatment level (*p* < 0.05).

To assess the survival time of EBL cells following infection with *M. bovis*, the cells were infected at different MOI and monitored for changes in viability at 24 h, 48 h, and 72 h post-infection using the CCK-8 kit. As shown in Figure 1, there was no statistically significant difference in the survival rate of EBL cells after 24 h infection with *M. bovis* across the different MOI. However, the survival rate of cells in the infected group at 48 hpi was significantly lower than that of the control group, with a reduction of approximately 20% observed in the group infected at 250 MOI. In contrast, the survival rate of cells within the infection group at 72 hpi exhibited a gradual decline correlating with the increase in *M. bovis* infection, with the survival rate in the 1000 MOI group dropping to 50%. Based on the results, the infection ratio of *M. bovis* to EBL in this study was set as 250, and considering the significant decrease in cell survival after 48 h of infection, subsequent experimental time points were established at 12, 24, and 48 h.

### 2.2. Oxidation Damage of EBL Cells Caused by M. bovis Infection

**Figure 2:**
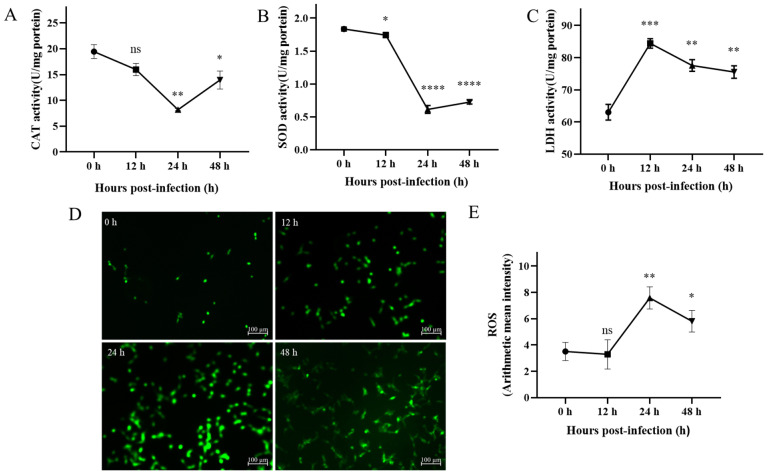
The related indexes of oxidative stress in EBL cells after 12 h, 24 h, and 48 h infection with *M. bovis*. (**A**) CAT levels at different time periods (12 hpi, 24 hpi, 48 hpi). (**B**) SOD levels at different time periods (12 hpi, 24 hpi, 48 hpi). (**C**) LDH levels at different time periods (12 hpi, 24 hpi, 48 hpi). (**D**) ROS level was detected by DCFH-DA staining fluorescence microscopy at different time periods (12 hpi, 24 hpi, 48 hpi). (**E**) Green fluorescence intensity was analyzed by image J. Data are presented as mean ± standard deviation (*n* = 3). Infection group compared with control group, ns *p* > 0.05, * *p* < 0.05, ** *p* < 0.01, *** *p* < 0.001, **** *p* < 0.0001.

In order to detect the oxidation damage of EBL cells caused by *M. bovis* infection, the contents of catalase (CAT), superoxide dismutase (SOD), lactate dehydrogenase (LDH), and ROS levels in EBL cells after infection with *M. bovis* were determined. In comparison to the control group, the activities of CAT and SOD were diminished in EBL cells infected with *M. bovis*. Specifically, CAT activity exhibited a significant reduction at 24 hpi (Figure 2A), while SOD activity significantly decreased at 12 hpi, reaching its lowest point at 24 hpi (Figure 2B). Conversely, LDH activity increased following *M. bovis* infection, attaining a significant level at 12 hpi (Figure 2C). In addition, green fluorescence was observed by fluorescence microscopy after staining with DCFH-DA. Compared with the uninfected group, green fluorescence was enhanced in EBL cells after infection with *M. bovis* (Figure 2D). Image J (version 2.14.0/1.54f) analysis of fluorescence intensity showed that ROS content in EBL cells reached a significant level at 24 hpi with *M. bovis* (Figure 2E).

### 2.3. MMP of EBL Cells Decreased After Infection with M. bovis

**Figure 3:**
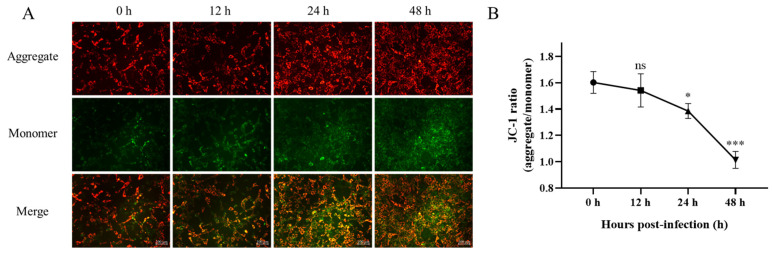
Effects of *M. bovis* on mitochondrial membrane potential in EBL cells. (**A**) EBL cells infected with *M. bovis* were collected, and the mitochondrial membrane potential was evaluated by JC-1 at different time periods (12 hpi, 24 hpi, 48 hpi). Aggregate: polymer (red light). Monomer; JC-1 monomer (green light). (**B**) Fluorescence intensity was analyzed by image J. Data are presented as mean ± standard deviation (*n* = 3). Infection group compared with control group, ns *p* > 0.05, * *p* < 0.05, *** *p* < 0.001.

In order to determine the relationship between the increase in ROS in EBL cells after *M. bovis* infection and mitochondrial damage. The EBL cells were treated with a JC-1 fluorescent probe according to the instructions after 12 h, 24 h, and 48 h with *M. bovis* infection. The alterations in MMP were assessed by observing and documenting the shifts in red and green fluorescence signals using fluorescence microscopy. As shown in Figure 3, when the MMP is high, JC-1 accumulates in the matrix of mitochondria and forms a polymer (red fluorescence). When the MMP is reduced, JC-1 cannot accumulate in the matrix of mitochondria, and JC-1 is a monomer (green fluorescence). Image J (version 2.14.0/1.54f) was used to analyze the average optical density of red and green fluorescence of cells, respectively, and calculate the ratio. The average fluorescence intensity analysis of Image J showed that compared with the control group, the ratio of red-green fluorescence in the infected group decreased and reached a significant level at 24 h, indicating a reduction in the MMP of EBL cells as a consequence of *M. bovis* infection.

### 2.4. Mitochondrial Structure Damage of EBL Cells Caused by M. bovis Infection

**Figure 4:**
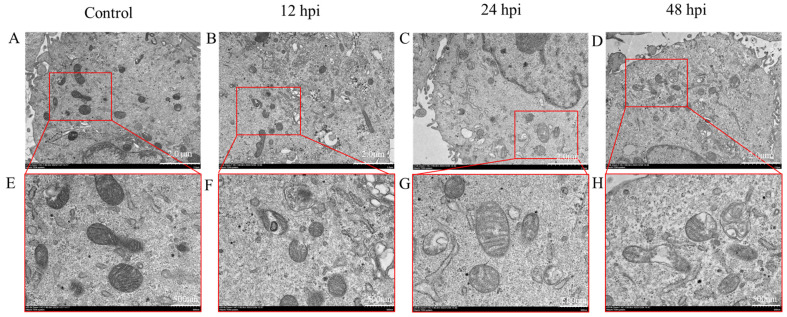
Effect of *M. bovis* on mitochondrial morphology in EBL cells. EBL cells infected with *M. bovis* at different time periods were collected and mitochondrial morphology was observed by transmission electron microscopy. (**A**,**E**) Mitochondrial morphology of uninfected group. (**B**,**F**) Mitochondrial morphology at 12 hpi. (**C**,**G**) Mitochondrial morphology at 24 hpi. (**D**,**H**) Mitochondrial morphology at 48 hpi (**A**–**D**) scale bar = 2 µm. (**E**–**H**) scale bar = 500 nm.

In order to further visualize the mitochondrial damage of EBL cells after *M. bovis* infection, transmission electron microscopy was used to observe the mitochondrial morphology of EBL cells in the control group and infected group (Figure 4). In the control group, the mitochondria exhibited a normal quantity, uniform distribution, intact membranes, orderly arrangement of cristae, and consistent electron density within the matrix. Conversely, in the *M. bovis* infection group, there was a notable reduction in mitochondrial numbers, irregular distribution, compromised membrane integrity, disorganized cristae arrangement, localized membrane damage, and a dissolved and diminished matrix. As the duration of infection increased, there was a progressive dissolution of the membrane in localized areas, accompanied by partial dissolution and leakage of the matrix.

### 2.5. EBL Cell Apoptosis Induced by M. bovis Infection

**Figure 5:**
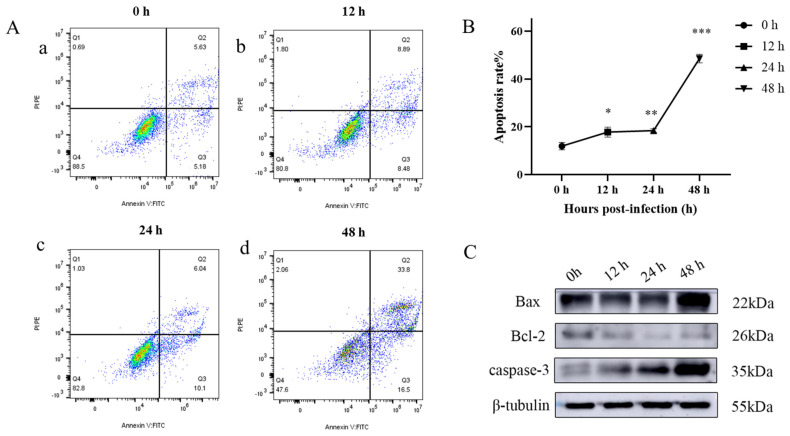
The apoptosis rate in EBL cells after 12 h, 24 h, and 48 h infection with *M. bovis*. (**A**) Apoptosis rate was detected by flow cytometry at 12 hpi, 24 hpi, and 48 hpi. (**a**) The apoptosis rate of uninfected EBL cells. (**b**–**d**) The apoptosis rates of *M. bovis*-infected cells at different time periods (12, 24, and 48 h). (**B**) The percentage of *M. bovis*-induced apoptosis in EBL cells with different times (12 hpi, 24 hpi, 48 hpi). (**C**) Apoptosis-related proteins were detected by Western Blotting at 12 hpi, 24 hpi, and 48 hpi. Data are the mean ± standard deviation of three independent experiments. Infection group compared with control group, ns *p* > 0.05, * *p* < 0.05, ** *p* < 0.01, *** *p* < 0.001.

In order to test whether *M. bovis* infection leads to mitochondrial pathway apoptosis, EBL cells were infected with *M. bovis* for 12 h, 24 h, and 48 h, and the apoptosis rate was detected by flow cytometry after Annexin V-FITC/PI staining. Annexin V-FITC/PI staining showed in Figure 5A, compared to uninfected cells, *M. bovis* was able to induce different degrees of apoptosis in EBL cells. With the increase in *M. bovis* infection time, the apoptosis rate of EBL cells increased from 20% to 50%. This increase attained statistical significance at 12 hpi (*p* < 0.05) and demonstrated a highly significant difference at 24 hpi (*p* < 0.001) (Figure 5B).

Western Blotting detected apoptotic protein caspase-3, pro-apoptotic protein Bax, and anti-apoptotic protein Bcl-2 in the Bcl-2 family, all of which are key regulators of mitochondria-related apoptotic factor release. Western Blotting results showed an upregulation in the pro-apoptotic protein Bax alongside an increase in the expression of the apoptotic protein caspase-3 following infection with *M. bovis*. Conversely, there was a down-regulation of the anti-apoptotic protein Bcl-2 (Figure 5C).

### 2.6. Identification of Proteins and Screening of Differentially Expressed Proteins

**Figure 6:**
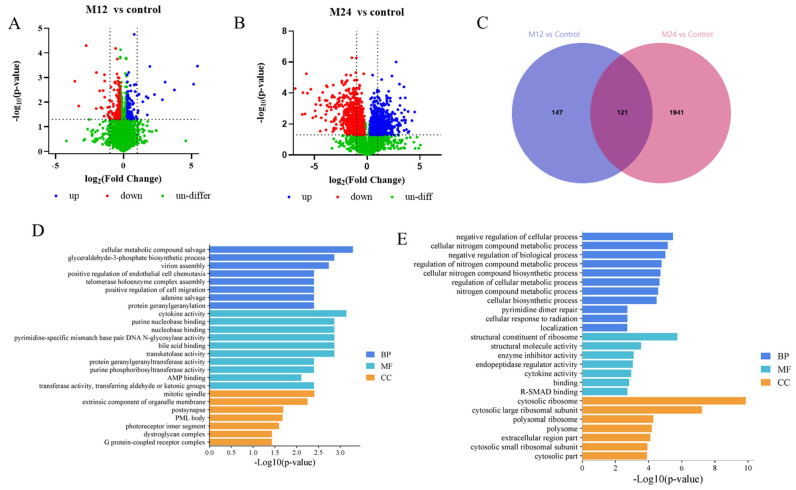
Proteomic data analysis. (**A**) Volcano map shows the DEPs at 12 hpi group. (**B**) Volcano map shows the DEPs at 24 hpi group. Blue-colored plots illustrate upregulated proteins, red-colored plots illustrate down-regulated proteins and green plots reflect proteins that did not show changes in expression. (**C**) Venn map shows the common up-down-regularized DEPs at 12 hpi and 24 hpi. (**D**) GO functional enrichment pathway of 12 hpi. (**E**) GO functional enrichment pathway of 24 hpi.

In order to screen host proteins associated with *M. bovis* infection, 4D-DIA proteomics was used to analyze EBL cells after *M. bovis* infection. Mass spectrometric analysis by DIA showed that the number of peptides was 76,341, and the number of proteins was 7222. The details of the peptide and protein information are provided in the Supplementary Materials. In this study, differentially expressed proteins (DEPs) were identified based on the criterion of (|Fold change| > 1.2) and significance level (*p* < 0.05). There were 268 DEPs in the 12 hpi group, of which 113 proteins were significantly upregulated, and 155 proteins were significantly down-regulated (Figure 6A). There were 2061 DEPs in the 24 hpi group, of which 1127 proteins were significantly upregulated and 935 proteins were significantly down-regulated (Figure 6B). There were 121 common DEPs between 12 hpi and 24 hpi, including 41 upregulated proteins and 66 down-regulated proteins (Figure 6C).

### 2.7. GO Analysis of DEP

In this study, the 280 and 764 the Gene Ontology (GO) terms were respectively annotated at 12 hpi and 24 hpi group. The details of the GO analysis are provided in the Supplementary Materials. The DEPs in the 12 hpi group were mainly involved in cellular metabolic compound salvage, glyceraldehyde-3-phosphate biosynthetic process, AMP binding, G protein-coupled receptor complex and mitotic spindle (Figure 6D). The DEPs in the 24 hpi group were mainly involved in the negative regulation of cellular process, regulation of nitrogen compound metabolic process, enzyme inhibitor activity, structural constituent of ribosome and cytosolic ribosome (Figure 6E).

### 2.8. KEGG Enrichment Analysis and PPI Network and Clustering Analyses of DEPs Associated with Mitochondrial Apoptosis

**Figure 7:**
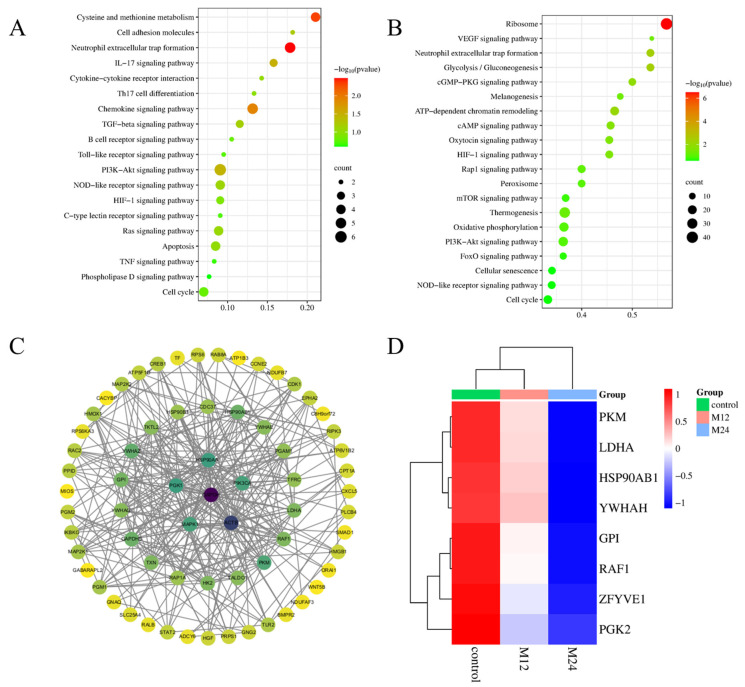
The enrichment analysis of selected DEPs in EBL cells infected with *M. bovis. (***A**) The KEGG enrichment analysis of selected DEPs at 12 hpi. (**B**) The KEGG enrichment analysis of selected DEPs at 24 hpi. (**C**) The interaction network map of important DEPs. (**D**) The heatmap of common DEPs cluster analysis.

In this study, we screened 150 KEGG signaling pathways in the 12 hpi group, and 234 KEGG signaling pathways in the 24 hpi group. The details of the KEGG analysis are provided in the Supplementary Materials. To explore the KEGG pathways linked to mitochondrial damage and apoptosis, we screened both 22 relevant signaling pathways in the 12 hpi and 24 hpi (Figure 7A,B). These pathways were mainly enriched in signal transduction, immune system, and cell growth and death pathways, including glycolysis/gluconeogenesis, pentose phosphate, oxidative phosphorylation, VEGF, RAP1, thermogenesis, AMPK, FoxO, cGMP-PKG, cAMP, calcium, Wnt, Phospholipase D, apoptosis, MAPK, cellular senescence, autophagy, cell cycle, Ras, PI3K-Akt, mTOR, HIF-1 signaling pathway.

Among the 22 signaling pathways associated with mitochondrial apoptosis shared by the 12 hpi and 24 hpi groups, there were 8 EBL cell proteins associated with *M. bovis* infection (Table 1). The differential proteins in the signaling pathway that may be involved in mitochondrial apoptosis induced by *M. bovis* infection were used to construct a protein interaction network diagram for the differentially expressed proteins based on the protein interaction relationship in the STRING database. Notably, proteins such as YWHAZ, PIK3CA, HSP90AB1, RAP1A, TXN, RAF1, MAPK1, PKM, PGK1, and GAPDH exhibited significant clustering within the PPI network, indicating their collaborative biological functions (Figure 7C). Furthermore, the expression levels of RAF1, PKM, YWHAH, HSP90AB1, and PGK2 were observed to decline in correlation with the increase in *M. bovis* time (Figure 7D).

## 3. Discussion

*M. bovis* infection has caused great economic losses to the cattle industry. In this study, the infection of *M. bovis* resulted in increased ROS levels, oxidative damage, decreased mitochondrial membrane potential and morphological damage, and increased apoptosis rate of EBL cells. ROS are the main free radicals in living organisms, with approximately 90% of ROS coming from the respiratory chain of the inner mitochondrial membrane [20]. Under normal physiological conditions, the antioxidant system of the body will remove ROS in time to maintain the balance between oxidation and antioxidants in the body. However, when the body is stimulated by different stressors or is infected by pathogenic bacteria, the electron leakage from the mitochondrial respiratory chain can produce superoxide free radicals, thereby increasing ROS levels. The excessive accumulation of ROS can result in membrane lipid peroxidation, which may cause structural damage to the cell membrane, mitochondrial membrane, and endoplasmic reticulum, ultimately leading to oxidative damage [21]. The mitochondria of the thymus tissue of chickens infected with *Mycoplasma gallisepticum* were swollen, and the chromatin material was concentrated. Concurrently, the expression of genes related to ATPase activity and energy metabolism was decreased in the thymus of *Mycoplasma gallisepticum*-infected chickens [22]. Furthermore, the internalization of *Staphylococcus aureus* into MAC-T cells has also been shown to elevate ROS levels and induce mitochondrial damage [23]. *M. bovis* destroys bMEC by generating ROS and activating a mitochondria-dependent apoptosis pathway, with varying degrees of damage observed among different isolates [24]. These findings align with the results of this study, suggesting *M. bovis* infection may lead to apoptosis of cells via the mitochondrial pathway.

In this study, a total of 268 and 2061 DEPs were identified at 12 h and 24 h of infection with *M. bovis*, respectively, utilizing the 4D-DIA technique. In earlier proteomic studies, the identity of calgranulin A and B was confirmed by MALDI-TOF/TOF MS. Calgranulin A and B were found in all patients infected with *Ureaplasma urealyticum* but not in any of the patients without infection, indicating that they are potential markers of intrauterine infection [25]. In contrast to the prior two-dimensional electrophoresis and mass spectrometry methods for identifying host proteins associated with mycoplasma infection, the 4D-DIA technology implemented in this study offers significant advantages, including enhanced scanning speed, increased sensitivity, and a notable improvement in both protein coverage depth and protein flux.

To investigate the mechanism of mitochondrial damage and apoptosis of EBL cells induced by *M. bovis* infection, we screened 22 KEGG pathways related to mitochondrial damage and apoptosis. *Vibrio splendidus* (*V. splendidus*) infection damaged mitochondrial morphology in coelomocytes and reduced mitochondrial membrane potential and mitophagosome formation; mitophagy activity is required for coelomocyte survival in *Apostichopus japonicus* against *V. splendidus* infection [20]. *Mycoplasma gallisepticum* infection triggers an inflammatory response mediated by the TLR-2/MyD88/NF-κB signaling pathway. This response results in decreased levels of autophagy and impaired energy metabolism, ultimately causing damage to thymic tissue in chickens [22]. In bovine pneumonia, *Staphylococcus aureus* triggers activate toll-like receptors (TLRs), which further elicit the activation of the inflammation via the NF-κB pathway, oxidative stress, and apoptotic pathways [23]. HeLa cells, after infection with *Mycoplasma agalactiae*, showed increased chromatin concentration and caspase-3 cleavage and apoptosis-like phenomenon [26]. *Mycoplasma hominis* infection causes damage to primary human keratinocytes, which affects the cell cycle by activating oxidative stress and toll-like receptor response pathways [27]. *Mycoplasma ovis* induces apoptosis of sheep epithelial cells by increasing the phosphorylated expression of p38 and pro-apoptotic proteins and activating caspase-3, caspase-8, and poly ADP-ribose polymerase cleavage [28]. Lipid-associated membrane protein (LAMP) of mycoplasma is the main pathogenic factor of mycoplasma disease and LAMP of *Mycoplasma suis pneumoniae* induces apoptosis of porcine alveolar macrophages through NO production, superoxide anion production, and caspase-3 activation [29]. This study is consistent with our results. Following infection with *M. bovis*, oxidative stress and mitochondrial damage lead to the apoptosis of EBL cells. Additionally, pathways associated with oxidative phosphorylation, MAPK signaling, toll-like receptors, and cell cycle regulation are implicated in this process; however, further investigation is required to elucidate the role of other signaling pathways.

In this study, we screened YWHAZ, PIK3CA, HSP90AB1, RAP1A, TXN, RAF1, MAPK1, PKM, PGK1, and GAPDH, which may play a role in the mitochondrial apoptotic pathway t activated by *M. bovis* infection. YWHAZ, also known as tyrosine 3 tryptophan 5 monooxygenase-activated protein, is one of the seven subtypes of 14-3-3 proteins involved in signal transduction, cell cycle regulation, apoptosis, and stress response [30]. miR-451, as a key factor regulating inflammation, directly targets YWHAZ to inhibit the proliferation and cell process of chicken embryo fibroblast (DF-1) cells infected with *Mycoplasma gallinarum* and induce cell apoptosis [31]. HSP90AB1 is a member of the heat shock protein family that maintains cell stability [32]. HSP90AB1 and its related homology HSP90AA1 have important potential in the treatment of lung cancer. HSP90AB1 can also mediate T-cell apoptosis through the Akt/SMARCC1/AP-1/ROS axis [33]. It also plays an important role in the process of autophagy induced by nervous necrosis virus invasion [34]. gga-miR-16-5p is upregulated in *Mycoplasma gallinarum*-infected DF-1 cells, and phosphoinosidine-3-kinase regulatory subunit 1 (PIK3R1) has been shown to be the target gene of gga-miR-16-5p. Overexpression of gga-miR-16-5p can down-regulate the protein expression of PIK3R1 and phosphorylated serine/threonine kinase (p-Akt), promoting apoptosis [35]. The PIK3CA gene also belongs to the PI3K/AKT signaling pathway, which can be activated by the activation of PIK3CA or the inactivation of PTEN [36]. PIK3CA overexpression of genes and their proteins after mutation, the AKT is continuously activated by PI3K/AKT pathways, thus inhibiting cell cycle and mitochondrial-mediated apoptosis [37]. Phosphorylated Akt also inhibits BAX-activated apoptosis by promoting hexokinase-2 (HK-2) translocation to mitochondria [38]. RAP1A, a small GTPase protein, has been identified as a new target of miR-501-3p. The overexpression of RAP1A significantly attenuates the inhibitory effect of miR-501-3p on the proliferation and motor ability of non-small cell lung cancer cells [39]. Rap1 consists of Rap1A and Rap1B subunits (subtypes); two Rap1 subtypes act through different signaling pathways. Rap1B is the major isomer necessary for normal VEGF-induced EC barrier dissolution [40]. Rap1B deficiency attenuates VEGF-induced permeability in vivo, and AJ remodeling in vitro Rap1B promotes VEGFR2 activation in endothelial cells via integrin α(v)β(3), thereby promoting VEGF-mediated angiogenesis [41]. DF-1 cells can exhibit resistance to *Mycoplasma gallinarum* infection through gga-miR-24-3p/RAP1B, which leads to reduced cellular proliferation and enhanced apoptosis [42]. The TXN system, composed of TXN, TXN reductase, and reduced Coenzyme II (NADPH), is one of the key mechanisms for maintaining intracellular REDOX balance [43]. The imbalance of TXN homeostasis will lead to chemical nerve injury, while the TXN system can treat chemical nerve injury by targeting active substances [44]. Inhibiting TXN expression has been shown to significantly decrease the proliferation, apoptosis, migration, and invasion of pancreatic cancer cells [45]. RAF1 can activate mitogen-activated protein kinase (MEK), then activate another serine/threonine kinase ERK, and activate the expression of downstream transcription factors to regulate cell proliferation, differentiation, and apoptosis [46]. MAPK1 (ERK) may act on the mitochondrial membrane, and after its activation, it can induce mitochondrial membrane depolarization and Cyt-C release, activate the caspase cascade, and induce mitochondrial apoptosis [47]. In addition, in the mitochondria-mediated apoptosis pathway, the activation of the EGFR-mediated Ras/Raf/MEK/ERK signaling cascade has been shown to enhance the release of cytochrome c into the cytoplasm. This process is associated with an upregulation of Bax expression and a down-regulation of Bcl-2 expression, ultimately leading to the activation of the caspase cascade [48]. Phosphoglycerate kinase 1 (PGK1), pyruvate kinase M (PKM), and glyceraldehyde-3-phosphate dehydrogenase (GAPDH) all play a role in the energy metabolism process of glycolysis [49]. Phosphoglycerate kinase 1 (PGK1) plays a crucial role in glycolysis, as it catalyzes the reversible transformation of 1,3-diphosphoglycerate into 3-phosphoglycerate, thereby facilitating ATP production. Pyruvate kinase M (PKM2), a key glycolytic enzyme, is involved in many cellular processes, including apoptosis. Intestinal epithelial PKM2 enhances cell survival under colitis conditions by activating Wnt/β-catenin signaling [50]. Furthermore, the upregulation of PKM2 may protect intestinal epithelial cells against apoptosis through Bcl-xl [51]. It is well known that glycolysis/gluconeogenesis, the pentose phosphate pathway, and oxidative phosphorylation coordinate to regulate mitochondrial energy metabolism. The AMPK signaling pathway is also involved as a cellular energy sensor, co-regulating cell fate [14]. In mammals, there exist two types of transmembrane adenylyl cyclase (tmAC) and soluble adenylyl cyclase (sAC). The sAC is a multi-subunit complex encoded by a single gene, primarily regulated by intracellular calcium ions and bicarbonate. The accumulation of Ca^2+^ within mitochondria activates intracellular sAC, leading to the production of cyclic adenosine monophosphate (cAMP), which subsequently activates protein kinase A (PKA). This activation results in the phosphorylation of proteins within the respiratory chain complex and the activation of downstream effector molecules, thereby regulating cellular physiological functions [52]. In conclusion, this study provides valuable data for understanding the response of mitochondria of EBL cells to *M. bovis* infection.

## 4. Materials and Methods

### 4.1. Strains and Culture Conditions

*M. bovis* strain was isolated from the lung tissue of dead cattle suspected of *M. bovis* disease in Wuwei City, Gansu Province, and was preserved by the Laboratory of Infectious Diseases of Gansu Agricultural University [53]. *M. bovis* was cultured in complete medium containing mycoplasma base medium, sodium pyruvate, and penicillin at 37 °C 5% CO_2_ incubator until it reached the logarithmic growth stage. Subsequently, the precipitate of *M. bovis* was collected via centrifuge at 4 °C 12,000× *g* for 5 min and then suspended with DMEM for future applications.

### 4.2. Embryonic Bovine Lung Cells Culture

EBL cells were cultured in high-glucose DMEM medium containing 10% fetal bovine serum (FBS) and subsequently enumerated using a cell counter following digestion with pancreatic enzymes. In all experimental procedures, EBL cells were maintained in 6-well or 96-well culture plates for a duration of 24 h, achieving a growth density of 70–80% in preparation for *M. bovis* infection.

### 4.3. Determination of Optimal Infection Dose of Mycoplasma bovis

After adjusting the concentration of *M. bovis*, EBL cells were subjected to infection with *M. bovis* of different MOI. Subsequent to different infection times, the cell survival rate was assessed in accordance with CCK-8 instructions (Biosharp, Wuhan, China). This process facilitated the identification of the optimal *M. bovis* infection ratio and provided a preliminary estimation of the appropriate infection time. MOI is defined as the ratio of the product of *M. bovis* concentration and bacterial fluid volume to the number of cells.

### 4.4. Antioxidant Capacity

EBL cells were subjected to infection with *M. bovis* at different times, and cell precipitation was collected at each time point. The extraction solution was subsequently added for cell fragmentation, and the supernatant was obtained following centrifugation at 8000× *g* for 10 min at 4 °C. The activities of antioxidant enzymes in the EBL cells were assessed using SOD, CAT, and LDH assay kits (Solarbio, Beijing, China) in conjunction with a spectrophotometer (Gene Quant 1300, GE, Boston, MA, USA), according to the protocols provided by the manufacturer. EBL cells were cultured in 6-well plates, with uninfected cells serving as the control group. Both the control and infected groups were maintained under identical culture conditions.

### 4.5. Reactive Oxygen Species (ROS)

The ROS kit (Servicebio, Wuhan, China) is based on the detection of ROS levels using a 2′,7′-dichlorofluorescein diacetate fluorescent probe (DCFH-DA). The EBL cells cultured in 6-well plates were subjected to infection with *M. bovis* for 12 h, 24 h, and 48 h. The levels of ROS were assessed using the ROS kit, with uninfected cells serving as controls. Following 12, 24, 48 h post-infection, EBL cells were washed with PBS and stained with the DCFH-DA working solution. The cells were then incubated at 37 °C for 30 min in a dark environment. Post-incubation, the DCFH-DA solution was removed, and the cells were washed 3 times with PBS buffer. The ROS levels were subsequently evaluated using an inverted fluorescence microscope (Zeiss Axio Scope A1, Carl Zeiss, Oberkochen, Germany) after the cells were covered in PBS.

### 4.6. Mitochondrial Membrane Potential (MMP)

MMP was assessed using the mitochondrial membrane potential detection kit (Solar-bio, Beijing, China). EBL cells infected with *M. bovis* were cultured in 6-well plates for 12 h, 24 h, and 48 h, with uninfected cells serving as controls. Following each time point, the cells were washed with PBS. Subsequently, 1 mL of cell culture medium was added, followed by the addition of 1 mL of the JC-1 staining working solution. The cells were then incubated at 37 °C for 20 min in the dark environment. After the incubation period, the supernatant was discarded, and the cells were washed with JC-1 staining buffer. Finally, 2 mL of cell culture medium was added, and the cells were examined using an inverted fluorescence microscope (Zeiss Axio Scope A1, Carl Zeiss, Germany).

### 4.7. The Ultrastructure of the Cells Was Observed by Transmission Electron Microscopy

The EBL cells were subjected to infection with *M. bovis* for 12 h, 24 h, and 48 h. Following the infection, the cell precipitation was obtained through centrifugation at 4 °C. The precipitation was fixed using a transmission electron microscope fixative (Servicebio, Wuhan, China) for a period of 24 h. After washing precipitation with 0.1 M phosphate buffer PB (PH 7.4), the precipitation was suspended and wrapped in the agarose. Dehydrate at room temperature using graded alcohol and acetone. Then, cells sequentially embedded in epoxy resin acetone mixtures (1:1) for 2 h, epoxy resin acetone mixtures (2:1) overnight at 37 °C, and then in pure resin for 5 h. After resin had polymerized, the resin blocks were cut down until 60-80 nm thin on the ultramicrotome (Leica, Wetzlar, Germany) and were fished out onto the 150-mesh cuprum grids. The cuprum grids were stained with 2% uranium acetate saturated alcohol solution and 2.6% lead citrate solution, and the cuprum grids were observed under Transmission Electron Microscope (Hitachi, Tokyo, Japan) and images were taken.

### 4.8. Apoptosis Rate

The Annexin V-FITC/PI Cell Apoptosis Detection Kit (Servicebio, Wuhan, China) was used to assess the apoptosis rate. The EBL cells were subjected to infection with *M. bovis* for 12 h, 24 h, and 48 h. Following this, the cultured supernatant was collected into a centrifuge tube, and then the cells were digested with pancreatic enzyme without EDTA. The digested cells were then transferred to a centrifuge tube corresponding to the collected supernatant, centrifuged at 400× *g* for 5 min, and subsequently washed and precipitated with PBS once. The cells were gently resuspended in 100 μL of 1 × Binding Buffer, mixed with 5 μL of Annexin V-FITC and 5 μL of PI at room temperature, and kept away from light for 8–10 min. Finally, the mixture was shaken with 400 µL of 1 × Binding Buffer in preparation for flow cytometry analysis (CytoFLEX, Beckman Coulter, Brea, CA, USA).

### 4.9. Determination of Mitochondrial Apoptosis-Related Proteins

The EBL cells were infected with *M. bovis* for 12 h, 24 h, and 48 h, and the cell precipitation was collected to extract the total protein of the cells according to Wu’s method [53]. SDS-PAGE gel electrophoresis was carried out with 20 μg as sample size, wet transfer was carried at 100 V voltage for 0.5–1 h, and 5% skim milk powder was enclosed in room temperature shaking table for 120 min. The PVDF membranes were incubated with anti-Bcl2 (1:2000, UpingBio, Hangzhou, China), anti-Bax (1:2000, UpingBio, Hangzhou, China), anti-Caspase-3 (1:2000, UpingBio, Hangzhou, China), and anti-beta-tubulin (1:3000, Bioss, Beijing, China). These were incubated at 4 °C overnight. Following this incubation, the membranes were washed three times with PBST for 10 min each. They were then incubated with anti-rabbit (1:8000, Affinity Biosciences, Cincinnati, OH, USA) or anti-mouse (1:3000, Bioss, Beijing, China) at room temperature for 90 min. Afterward, the membranes were washed again with PBST 3 times for 10 min each time; detection was performed using ECL chemiluminescence reagent (Beyotime, Shanghai, China) in conjunction with a chemiluminescence detection instrument (GE, MA, USA).

### 4.10. Processing and Preparation of Proteomic Test Samples

EBL cells were infected with *M. bovis* for 12 h and 24 h, with uninfected cells serving as the control group. Cell precipitation was collected, and three replicates were collected in each group. Appropriate amount of SDT (4% SDS, 100 mM Tris-HCl, pH 7.6) was added to each sample to extract protein, and the protein was quantified by BCA method. A total of 20 µg protein was taken from each sample and added to an appropriate amount of 5× loading buffer, then bathed in boiling water for 5 min for SDS-PAGE electrophoresis. Appropriate amount of protein was taken from all samples and mixed into pool samples for the construction of spectral library. All samples were enzymolysis by filter-aided proteome preparation (FASP). The peptide segment of the enzymatic pool sample was graded using Thermo’s high-pH-inverted peptide separation kit (Thermo Fisher Scientific, Waltham, MA, USA). The classified pool sample peptides and the peptide segments of all the remaining samples are desalinated using C18 Cartridge. The peptide was lyophilized and redissolved in 40 μL 0.1% formic acid solution. The peptide concentration was determined by OD 280. Appropriate amount of iRT standard peptide was added to pool sample peptide and enzymolysis peptide of each sample, respectively, and DDA mass spectrometry and DIA mass spectrometry were performed.

### 4.11. DEPs Identification

For the analysis of DDA library data, the FASTA sequence database was searched with Spectronaut software(version Spectronaut^TM^ 14.4.200727.47784). The database was downloaded at the following website: http://www.uniprot.org (accessed on 18 June 2024). Additionally, iRT peptides sequence was added (Biognosys|iRT Kit|: Omicsolution, Shanghai, China). The parameters were set as follows: enzyme is trypsin, max missed cleavages is 1, fixed modification is carbamidomethyl (C), dynamic modification is oxidation (M) and acetyl (Protein N-term). All reported data were based on 99% confidence for protein identification as determined by false discovery rate (FDR) ≤ 1%.

DIA data were analyzed with Spectronaut^TM^ 14.4.200727.47784, searching the above constructed spectral library. Main software parameters were set as follows: retention time prediction type is dynamic iRT, interference on MS2 level correction is enabled, and cross-run normalization is enabled. All results were filtered based on Q value cutoff of 0.01 (equivalent to FDR < 1%).

### 4.12. Bioinformatic Analysis of Differentially Expressed Proteins

GO analysis of DEPs was carried out using the software program Blast2GO(version BLASTP 2.8.0+). Fisher’s Exact Test was used to compare the distribution of each GO classification in the target protein set, and the enrichment analysis of GO annotation was performed on the target protein set. Signaling pathway analysis was performed with the KEGG database (http://www.genome.jp/kegg/pathway.html, accessed on 10 July 2024). Fisher’s Exact Test was used to compare the distribution of KEGG pathway in the target protein set, and the enrichment analysis of KEGG pathway annotation was performed on the target protein set. Based on STRING (http://string-db.org/, accessed on 9 August 2024), the PPI networks (confidence score ≥ 0.40) of DEPs were obtained. The results were downloaded in the XGMML format and imported into Cytoscape software (http://www.cytoscape.org/, version 3.2.1) to visualize and further analyze functional protein–protein interaction networks.

### 4.13. Statistical Analysis

All the experiments were performed in triplicate, and the representative data were obtained from three independent experiments. Statistical analysis was carried out utilizing GraphPad Prism 8. A one-way analysis of variance (ANOVA) was used to identify statistical differences between the control group and the infected group.

## 5. Conclusions

*M. bovis* infection resulted in increased ROS levels, oxidative damage, decreased mitochondrial membrane potential and morphological damage, and increased apoptosis rate of EBL cells. The proteome analysis identified DEPs and 22 relevant signaling pathways associated with mitochondrial damage and apoptosis. In addition, YWHAZ, PIK3CA, HSP90AB1, RAP1A, TXN, RAF1, MAPK1, PKM, PGK1, and GAPDH might be involved in mitochondrial pathway apoptosis induced by *M. bovis* infection. This proteomics study lays the groundwork for subsequent research into the between-host proteins and *M. bovis* infection.

## Figures and Tables

**Table 1:** Response of EBL cells to *M. bovis* infection—signaling pathways associated with mitochondrial apoptosis.

Number	KEGG Pathway	Specific Proteins		Common Proteins
		12 hpi	24 hpi	
1	Glycolysis/Gluconeogenesis		PGM2/HK2/GAPDH/PGM1/GAPDHS/PGAM1/PGK1	PKM/LDHA/PGK2/GPI
2	Pentose phosphate pathway	TKTL2	PGM2/TALDO1/PGM1/Q2HJ58/	GPI
3	cGMP-PKG signaling pathway	PLCB4	ADCY6/GNAQ/SLC25A4/CREB1/MAPK1/MAP2K1/MAP2K2/ATP1B3	RAF1
4	cAMP signaling pathway		ORAI1/RAF1/RAC2/ADCY6/CREB1/PIK3CA/MAPK1/RAP1A/MAP2K1/MAP2K2/ATP1B3	RAF1
5	HIF-1 signaling pathway	TF	TFRC/HK2/GAPDH/PIK3CA/MAPK1/MAP2K1/MAP2K2/PGK1/RPS6/HMOX1	LDHA/PGK2
6	VEGF signaling pathway		RAC2/PIK3CA/MAPK1/MAP2K1/MAP2K2	RAF1
7	Thermogenesis	PLCB4	WNT5B/ADCY6/GNAQ/CREB1/MAPK1/MAP2K1/MAP2K2	
8	PI3K-Akt signaling pathway	ATP5F1D/NDUFB7/NDUFAF3	CPT1A	
9	Oxidative phosphorylation	TLR2/GNG2/IKBKG	HGF/YWHAG/EPHA2/CREB1/PIK3CA/MAPK1/YWHAE/YWHAZ/MAP2K1/MAP2K2/RPS6/CCNE2/CDC37/HSP90AA1/HSP90B1	
10	Rap1 signaling pathway	IKBKG	IL6ST/MAPK1/HSP90AA1	HSP90AB1
11	mTOR signaling pathway	PLCB4	HGF/RALB/RAC2/ADCY6/GNAQ/EPHA2/PIK3CA/MAPK1/ACTB/RAP1A/MAP2K1/MAP2K2	RAF1
12	Calcium signaling pathway		WNT5B/RPS6KA3/MIOS/ATP6V1B2PIK3CA/MAPK1/MAP2K1/MAP2K2/CAB39/RPS6	
13	FoxO signaling pathway	SRGAP2	BMPR2/WNT5B/NTN4/CFL1/RAC2/EPHA2/PIK3CA/MAPK1/CDK5	RAF1
14	Cell cycle	PLCB4	HGF/ORAI1/GNAQ/SLC25A4	
15	Cellular senescence	BUB1B/YWHAH/HDAC2/CDC26	YWHAG/CDK1/YWHAE/YWHAZ/YWHAH/TFDP1/PLK1/CCNE2	
16	Phospholipase D signaling pathway	PLCB4/TXN/HSP90AB1/IKBKG	RIPK3/STAT2/TXN/MAPK1/GABARAPL2/YWHAE/HSP90AB1/HSP90AA1	
17	Wnt signaling pathway		SLC25A4/PPID/PIK3CA/MAPK1/CDK1/MAP2K1/MAP2K2/CCNE2	
18	AMPK signaling pathway	PLCB4	WNT5B/RAC2/CACYBP	
19	MAPK signaling pathway	PLCB4/GNG2	ADCY6/GNAQ/MAPK1/MAP2K1/MAP2K2/RPS6	RAF1
20	Autophagy-animal	SMAD1	BMPR2/WNT5B/YWHAG/ACTB/YWHAE/YWHAZ	YWHAH
21	Ras signaling pathway	CXCL5/IKBKG	MAPK1/HSP90AA1/HSP90B1	HSP90AB1
22	Apoptosis	RAB8A	C9orf72/HMGB1/PIK3CA/MAPK1/GABARAPL2/MAP2K1/MAP2K2/YKT6	RAF1/ZFYVE1

## Data Availability

All the research data are shared in this manuscript.

## Supplementary Materials

The following supporting information can be downloaded at: https://www.mdpi.com/article/10.3390/ijms26020823/s1.
