# Supplementary material for: Proteomics Reveals the Response Mechanism of Embryonic Bovine Lung Cells to Mycoplasma bovis Infection
Source: Int J Mol Sci. 2025 Jan 19;26(2):823. doi: 10.3390/ijms26020823 (PMC11765741; doi:10.3390/ijms26020823)
Supplement: Supplementary file 1 [file ijms-26-00823-s001.zip › GO.pdf]

Number	GO Term(12 hpi)	Category	P value
1	cellular metabolic compound salvage	P	0.000514
2	cytokine activity	F	0.000725
3	glyceraldehyde-3-phosphate biosynthetic process	P	0.001372
4	purine nucleobase binding	F	0.001372
5	nucleobase binding	F	0.001372
6	pyrimidine-specific mismatch base pair DNA N-glycosylase activity	F	0.001372
7	bile acid binding	F	0.001372
8	transketolase activity	F	0.001372
9	virion assembly	P	0.001831
10	protein dimerization activity	F	0.003564
11	mitotic spindle	C	0.003957
12	protein geranylgeranyltransferase activity	F	0.004015
13	positive regulation of endothelial cell chemotaxis by VEGF-activated vascular endothelial growth factor receptor signaling pathway	P	0.004015
14	telomerase holoenzyme complex assembly	P	0.004015
15	protein geranylgeranylation	P	0.004015
16	purine phosphoribosyltransferase activity	F	0.004015
17	transferase activity, transferring aldehyde or ketonic groups	F	0.004015
18	positive regulation of cell migration by vascular endothelial growth factor signaling pathway	P	0.004015
19	adenine salvage	P	0.004015
20	mismatch base pair DNA N-glycosylase activity	F	0.004015
21	DNA catabolic process, exonucleolytic	P	0.004015
22	purine-containing compound salvage	P	0.004995
23	regulation of steroid biosynthetic process	P	0.004995
24	positive regulation of sprouting angiogenesis	P	0.004995
25	viral budding	P	0.004995
26	extrinsic component of organelle membrane	C	0.005614
27	regulation of cholesterol biosynthetic process	P	0.007834
28	AMP binding	F	0.007834
29	positive regulation of CREB transcription factor activity	P	0.007834
30	adenine metabolic process	P	0.007834
31	regulation of sterol biosynthetic process	P	0.007834
32	purine nucleobase salvage	P	0.007834
33	adenine biosynthetic process	P	0.007834
34	aldehyde biosynthetic process	P	0.007834
35	lipid droplet organization	P	0.008666
36	endoplasmic reticulum calcium ion homeostasis	P	0.008666
37	regulation of peptidyl-serine phosphorylation	P	0.009957
38	sulfur compound biosynthetic process	P	0.011173
39	cellular response to endogenous stimulus	P	0.01176

40	NAD-dependent histone deacetylase activity (H3-K14 specific)	F	0.012738
41	primary miRNA binding	F	0.012738
42	positive regulation of endothelial cell chemotaxis	P	0.012738
43	regulation of alcohol biosynthetic process	P	0.012738
44	T cell mediated cytotoxicity	P	0.012738
45	amino acid salvage	P	0.012738
46	DNA clamp loader activity	F	0.012738
47	protein-DNA loading ATPase activity	F	0.012738
48	L-methionine salvage	P	0.012738
49	negative regulation of steroid metabolic process	P	0.012738
50	negative regulation of steroid biosynthetic process	P	0.012738
51	histone deacetylase activity (H3-K14 specific)	F	0.012738
52	mRNA methylation	P	0.012738
53	methionine biosynthetic process	P	0.012738
54	L-methionine salvage from methylthioadenosine	P	0.012738
55	purine ribonucleoside salvage	P	0.012738
56	L-methionine biosynthetic process	P	0.012738
57	positive regulation of peptidyl-serine phosphorylation	P	0.014556
58	positive regulation of telomerase activity	P	0.016509
59	SUMO binding	F	0.018642
60	IMP salvage	P	0.018642
61	SREBP signaling pathway	P	0.018642
62	DNA N-glycosylase activity	F	0.018642
63	regulation of endothelial cell chemotaxis	P	0.018642
64	protein prenylation	P	0.018642
65	positive regulation of protein depolymerization	P	0.018642
66	cellular response to sterol depletion	P	0.018642
67	prenylation	P	0.018642
68	purine nucleotide salvage	P	0.018642
69	positive regulation of actin filament depolymerization	P	0.018642
70	protein prenyltransferase activity	F	0.018642
71	protein kinase C binding	F	0.019772
72	regulation of steroid metabolic process	P	0.019772
73	protein localization to chromosome, telomeric region	P	0.019772
74	regulation of telomerase activity	P	0.019772
75	regulation of angiogenesis	P	0.019865
76	postsynapse	C	0.020464
77	cation binding	F	0.020988
78	PML body	C	0.021208
79	positive regulation of angiogenesis	P	0.021208
80	purine-containing compound biosynthetic process	P	0.022946
81	cellular response to hormone stimulus	P	0.023443
82	response to endogenous stimulus	P	0.024169
83	purine nucleoside monophosphate biosynthetic process	P	0.025136

84	purine ribonucleoside monophosphate biosynthetic process	P	0.025136
85	mRNA modification	P	0.025465
86	sulfur amino acid biosynthetic process	P	0.025465
87	nuclear body organization	P	0.025465
88	response to sterol depletion	P	0.025465
89	intramolecular oxidoreductase activity, interconverting aldoses and ketoses	F	0.025465
90	regulation of cholesterol metabolic process	P	0.025465
91	photoreceptor inner segment	C	0.025465
92	positive regulation of vasculature development	P	0.026527
93	regulation of vasculature development	P	0.02671
94	regulation of sprouting angiogenesis	P	0.027285
95	cellular response to growth factor stimulus	P	0.028168
96	small molecule biosynthetic process	P	0.030054
97	response to growth factor	P	0.03077
98	receptor ligand activity	F	0.032577
99	NAD-dependent histone deacetylase activity	F	0.033132
100	NAD-dependent protein deacetylase activity	F	0.033132
101	coronary vasculature development	P	0.033132
102	midbody abscission	P	0.033132
103	alkali metal ion binding	F	0.033132
104	monocarboxylic acid binding	F	0.033132
105	transmembrane receptor protein serine/threonine kinase signaling pathway	P	0.033848
106	purine ribonucleotide biosynthetic process	P	0.035242
107	organic acid biosynthetic process	P	0.035484
108	heparin binding	F	0.036108
109	response to molecule of bacterial origin	P	0.036879
110	metal ion binding	F	0.036905
111	5-aminolevulinate synthase activity	F	0.037109
112	antigen processing and presentation of endogenous peptide antigen via MHC class I via ER pathway, TAP-dependent	P	0.037109
113	positive regulation of maintenance of permeability of blood-brain barrier	P	0.037109
114	positive regulation of respiratory burst	P	0.037109
115	titin binding	F	0.037109
116	antigen processing and presentation of exogenous protein antigen via MHC class Ib, TAP-dependent	P	0.037109
117	dystroglycan complex	C	0.037109
118	dol-P-Man:Man(5)GlcNAc(2)-PP-Dol alpha-1,3-mannosyltransferase activity	F	0.037109
119	positive regulation of peptidyl-cysteine S-nitrosylation	P	0.037109
120	N-terminal protein N-methyltransferase activity	F	0.037109
121	rRNA (cytosine-N4-)-methyltransferase activity	F	0.037109

122	G protein-coupled receptor complex	C	0.037109
123	sensory perception of taste	P	0.037109
124	oxidoreductase activity, acting on paired donors, with incorporation or reduction of molecular oxygen, reduced ascorbate as one donor, and incorporation of one atom of oxygen	F	0.037109
125	mitotic DNA replication maintenance of fidelity	P	0.037109
126	positive regulation of positive chemotaxis	P	0.037109
127	O-phospho-L-serine:2-oxoglutarate aminotransferase activity	F	0.037109
128	cell cycle DNA replication maintenance of fidelity	P	0.037109
129	maintenance of permeability of blood-brain barrier	P	0.037109
130	mitotic recombination-dependent replication fork processing	P	0.037109
131	chondroitin-glucuronate 5-epimerase activity	F	0.037109
132	positive regulation of cholesterol metabolic process	P	0.037109
133	UDP-alpha-D-glucose:glucosyl-glycogenin alpha-D-glucosyltransferase activity	F	0.037109
134	activation of adenylate cyclase activity	P	0.037109
135	titin Z domain binding	F	0.037109
136	sensory perception of umami taste	P	0.037109
137	death-inducing signaling complex assembly	P	0.037109
138	acireductone dioxygenase [iron(II)-requiring] activity	F	0.037109
139	sterol response element binding	F	0.037109
140	maintenance of animal organ identity	P	0.037109
141	linear polyubiquitin binding	F	0.037109
142	regulation of heart rate by chemical signal	P	0.037109
143	negative regulation of collateral sprouting in absence of injury	P	0.037109
144	exon-exon junction complex disassembly	P	0.037109
145	inositol 1,4,5-trisphosphate receptor activity involved in regulation of postsynaptic cytosolic calcium levels	F	0.037109
146	histone H4-K8 acetylation	P	0.037109
147	negative regulation of vitamin D receptor signaling pathway	P	0.037109
148	N-terminal peptidyl-proline methylation	P	0.037109
149	negative regulation of transforming growth factor beta activation	P	0.037109
150	positive regulation of peptidyl-tyrosine autophosphorylation	P	0.037109
151	glucocorticoid catabolic process	P	0.037109
152	G/U mismatch-specific uracil-DNA glycosylase activity	F	0.037109
153	regulation of maintenance of permeability of blood-brain barrier	P	0.037109
154	ATP citrate synthase activity	F	0.037109
155	transforming growth factor beta receptor activity, type II	F	0.037109
156	antigen processing and presentation of endogenous peptide antigen via MHC class Ib via ER pathway, TAP-dependent	P	0.037109
157	positive regulation of neuroinflammatory response	P	0.037109
158	peptidyl-alanine modification	P	0.037109
159	regulation of testosterone biosynthetic process	P	0.037109
160	N-acylneuraminate-9-phosphate synthase activity	F	0.037109

161	platelet dense granule membrane	C	0.037109
162	ribonuclease III complex	C	0.037109
163	N-terminal peptidyl-alanine trimethylation	P	0.037109
164	sarcoglycan complex	C	0.037109
165	negative regulation of intrinsic apoptotic signaling pathway in response to osmotic stress	P	0.037109
166	regulation of positive chemotaxis	P	0.037109
167	omegasome	C	0.037109
168	anterograde axonal protein transport	P	0.037109
169	eukaryotic initiation factor 4G binding	F	0.037109
170	VEGF-activated platelet-derived growth factor receptor signaling pathway	P	0.037109
171	RNA folding	P	0.037109
172	vascular endothelial growth factor receptor 1 binding	F	0.037109
173	phospholipase C-activating angiotensin-activated signaling pathway	P	0.037109
174	regulation of collateral sprouting in absence of injury	P	0.037109
175	taurine biosynthetic process	P	0.037109
176	cap2 mRNA methylation	P	0.037109
177	platelet dense granule	C	0.037109
178	histone H4-K5 acetylation	P	0.037109
179	microtubule-severing ATPase activity	F	0.037109
180	regulation of protein kinase D signaling	P	0.037109
181	mitochondrion migration along actin filament	P	0.037109
182	dendrite membrane	C	0.037109
183	alkanesulfonate biosynthetic process	P	0.037109
184	CDP-diacylglycerol-glycerol-3-phosphate 3-phosphatidyltransferase activity	F	0.037109
185	oxalate transmembrane transporter activity	F	0.037109
186	Rad17 RFC-like complex	C	0.037109
187	histone H4-K12 acetylation	P	0.037109
188	microprocessor complex	C	0.037109
189	negative regulation of secondary metabolite biosynthetic process	P	0.037109
190	negative regulation of melanin biosynthetic process	P	0.037109
191	positive regulation of cholesterol biosynthetic process	P	0.037109
192	N-terminal peptidyl-alanine methylation	P	0.037109
193	protein localization to presynapse	P	0.037109
194	antigen processing and presentation of endogenous peptide antigen via MHC class Ib via ER pathway	P	0.037109
195	secretory granule localization	P	0.037109
196	positive regulation of alcohol biosynthetic process	P	0.037109
197	N-terminal peptidyl-serine methylation	P	0.037109
198	keto-deoxynonulosonic acid (KDN) cytidylyltransferase activity	F	0.037109
199	protein localization to nuclear body	P	0.037109

200	protein kinase C inhibitor activity	F	0.037109
201	N-terminal peptidyl-serine trimethylation	P	0.037109
202	epithelial fluid transport	P	0.037109
203	membrane depolarization during action potential	P	0.037109
204	N-acylneuraminate cytidylyltransferase activity	F	0.037109
205	transcription factor TFIIE complex	C	0.037109
206	positive regulation of sterol biosynthetic process	P	0.037109
207	extrinsic component of omegasome membrane	C	0.037109
208	regulation of histone H3-K36 methylation	P	0.037109
209	telomere formation via telomerase	P	0.037109
210	N-glycolylneuraminic acid (Neu5Gc) cytidylyltransferase activity	F	0.037109
211	antigen processing and presentation of exogenous peptide antigen via MHC class Ib	P	0.037109
212	protein localization to Cajal body	P	0.037109
213	glucose-6-phosphate isomerase activity	F	0.037109
214	methylthioribulose 1-phosphate dehydratase activity	F	0.037109
215	adenine binding	F	0.037109
216	unconventional myosin complex	C	0.037109
217	generation of catalytic spliceosome for second transesterification step	P	0.037109
218	N-terminal peptidyl-serine dimethylation	P	0.037109
219	antigen processing and presentation of endogenous peptide antigen via MHC class I via ER pathway	P	0.037109
220	recombinase activity	F	0.037109
221	N-succinyltransferase activity	F	0.037109
222	post-spliceosomal complex	C	0.037109
223	CAAX-protein geranylgeranyltransferase complex	C	0.037109
224	omegasome membrane	C	0.037109
225	UDP-sugar diphosphatase activity	F	0.037109
226	negative regulation of cyclic-nucleotide phosphodiesterase activity	P	0.037109
227	N-terminal protein amino acid methylation	P	0.037109
228	negative regulation of testosterone biosynthetic process	P	0.037109
229	positive regulation of cell proliferation by VEGF-activated platelet derived growth factor receptor signaling pathway	P	0.037109
230	N-terminal peptidyl-proline dimethylation	P	0.037109
231	adenine phosphoribosyltransferase activity	F	0.037109
232	glycoprotein transport	P	0.037109
233	FATZ binding	F	0.037109
234	glycogenin glucosyltransferase activity	F	0.037109
235	myosin V complex	C	0.037109
236	positive regulation of protein kinase D signaling	P	0.037109
237	triose-phosphate isomerase activity	F	0.037109
238	Golgi vesicle docking	P	0.037109
239	aryl hydrocarbon receptor complex	C	0.037109

240	photoreceptor cell maintenance	P	0.037109
241	bile acid transmembrane transporter activity	F	0.037109
242	testosterone biosynthetic process	P	0.037109
243	extension of a leading process involved in cell motility in cerebral cortex radial glia guided migration	P	0.037109
244	positive regulation of microglial cell activation	P	0.037109
245	angiogenin-PRI complex	C	0.037109
246	N-terminal peptidyl-glycine methylation	P	0.037109
247	viral life cycle	P	0.037833
248	identical protein binding	F	0.038482
249	positive regulation of response to external stimulus	P	0.039698
250	protein homodimerization activity	F	0.039815
251	sulfur compound metabolic process	P	0.040802
252	purine nucleotide biosynthetic process	P	0.040802
253	methionine metabolic process	P	0.041572
254	vascular endothelial growth factor receptor signaling pathway	P	0.041572
255	cellular response to interleukin-4	P	0.041572
256	vascular endothelial growth factor signaling pathway	P	0.041572
257	aspartate family amino acid biosynthetic process	P	0.041572
258	negative regulation of DNA replication	P	0.041572
259	nucleoside salvage	P	0.041572
260	ribonucleoside monophosphate biosynthetic process	P	0.042116
261	transforming growth factor beta receptor signaling pathway	P	0.042644
262	ATPase activity, coupled	F	0.042778
263	glycolytic process	P	0.043047
264	pyruvate biosynthetic process	P	0.043047
265	ATP generation from ADP	P	0.043047
266	I band	C	0.046215
267	maintenance of protein localization in organelle	P	0.046215
268	regulation of actin filament depolymerization	P	0.046215
269	pigment biosynthetic process	P	0.046215
270	DNA-dependent ATPase activity	F	0.046215
271	serine-type endopeptidase inhibitor activity	F	0.046215
272	Z disc	C	0.046215
273	cellular component assembly involved in morphogenesis	P	0.046215
274	serine-type endopeptidase activity	F	0.046215
275	Golgi stack	C	0.04691
276	response to decreased oxygen levels	P	0.04691
277	response to hypoxia	P	0.04691
278	active transmembrane transporter activity	F	0.049109
279	nucleoside monophosphate biosynthetic process	P	0.049109
280	positive regulation of macromolecule biosynthetic process	P	0.049896

Number	GO Term(24 hpi)	Category	P value
1	cytosolic ribosome	C	1.38E-10
2	cytosolic large ribosomal subunit	C	6.15E-08
3	structural constituent of ribosome	F	1.87E-06
4	negative regulation of cellular process	P	3.37E-06
5	cellular nitrogen compound metabolic process	P	7.01E-06
6	negative regulation of biological process	P	9.8E-06
7	regulation of nitrogen compound metabolic process	P	1.65E-05
8	cellular nitrogen compound biosynthetic process	P	1.91E-05
9	regulation of cellular metabolic process	P	2.2E-05
10	nitrogen compound metabolic process	P	2.67E-05
11	cellular biosynthetic process	P	3.31E-05
12	polysomal ribosome	C	5.25E-05
13	regulation of primary metabolic process	P	5.37E-05
14	organic substance metabolic process	P	5.56E-05
15	biosynthetic process	P	5.8E-05
16	metabolic process	P	6.22E-05
17	polysome	C	6.39E-05
18	organic substance biosynthetic process	P	6.48E-05
19	cellular process	P	6.54E-05
20	organonitrogen compound metabolic process	P	7.46E-05
21	cellular macromolecule biosynthetic process	P	7.46E-05
22	extracellular region part	C	7.96E-05
23	cytosolic small ribosomal subunit	C	0.000119
24	cytosolic part	C	0.000125
25	regulation of metabolic process	P	0.000129
26	regulation of macromolecule metabolic process	P	0.000138
27	DNA metabolic process	P	0.00014
28	cellular metabolic process	P	0.000143
29	regulation of biological process	P	0.000147
30	macromolecule biosynthetic process	P	0.000157
31	cytoplasmic translation	P	0.00019
32	multicellular organism development	P	0.000191
33	extracellular region	C	0.000203
34	ribosome	C	0.00021
35	negative regulation of proteolysis	P	0.000222
36	system development	P	0.000252
37	primary metabolic process	P	0.000273
38	structural molecule activity	F	0.000286
39	multicellular organismal process	P	0.00031
40	heterocycle metabolic process	P	0.00032
41	regulation of DNA metabolic process	P	0.000352
42	biological regulation	P	0.000366
43	cellular macromolecule metabolic process	P	0.000366

44	negative regulation of nitrogen compound metabolic process	P	0.000416
45	regulation of molecular function	P	0.000436
46	negative regulation of cellular metabolic process	P	0.000463
47	extracellular matrix	C	0.000482
48	vasculature development	P	0.00052
49	pigment granule	C	0.000547
50	melanosome	C	0.000547
51	negative regulation of cellular component organization	P	0.00057
52	developmental process	P	0.000575
53	macromolecule metabolic process	P	0.000612
54	extracellular space	C	0.000656
55	cardiovascular system development	P	0.000662
56	negative regulation of metabolic process	P	0.000712
57	somatic recombination of immunoglobulin gene segments	P	0.00075
58	cellular aromatic compound metabolic process	P	0.000752
59	regulation of cell differentiation	P	0.000797
60	enzyme inhibitor activity	F	0.0008
61	macromolecule localization	P	0.000803
62	anatomical structure development	P	0.000888
63	endopeptidase regulator activity	F	0.000906
64	removal of superoxide radicals	P	0.000922
65	animal organ morphogenesis	P	0.000925
66	vesicle targeting	P	0.000981
67	cellular amide metabolic process	P	0.00101
68	regulation of nucleobase-containing compound metabolic process	P	0.00101
69	negative regulation of macromolecule metabolic process	P	0.001074
70	regulation of intrinsic apoptotic signaling pathway in response to DNA damage	P	0.001099
71	response to organic substance	P	0.001129
72	cytokine activity	F	0.001131
73	blood vessel development	P	0.001149
74	animal organ development	P	0.001154
75	regulation of developmental process	P	0.001171
76	regulation of protein stability	P	0.001198
77	somatic diversification of immune receptors via germline recombination within a single locus	P	0.001209
78	somatic diversification of immunoglobulins	P	0.001209
79	somatic cell DNA recombination	P	0.001209
80	endosomal transport	P	0.001209
81	organic cyclic compound metabolic process	P	0.001218
82	regulation of cellular process	P	0.001285
83	cellular component organization	P	0.001304
84	muscle cell differentiation	P	0.001353
85	cellular component organization or biogenesis	P	0.001393

86	response to radiation	P	0.001415
87	response to ionizing radiation	P	0.001421
88	response to chemical	P	0.001461
89	spindle organization	P	0.001479
90	metaphase plate congression	P	0.001487
91	intrinsic apoptotic signaling pathway in response to DNA damage	P	0.001487
92	binding	F	0.001498
93	gene expression	P	0.001522
94	nucleobase-containing compound metabolic process	P	0.001525
95	peptide biosynthetic process	P	0.001569
96	amide biosynthetic process	P	0.001588
97	regulation of chromatin organization	P	0.001598
98	protein metabolic process	P	0.00165
99	somatic diversification of immune receptors	P	0.001738
100	cyclin-dependent protein kinase holoenzyme complex	C	0.001744
101	regulation of multicellular organismal development	P	0.001827
102	negative regulation of nucleobase-containing compound metabolic process	P	0.001861
103	regulation of astrocyte differentiation	P	0.001891
104	R-SMAD binding	F	0.001891
105	pyrimidine dimer repair	P	0.001891
106	cellular response to radiation	P	0.001907
107	localization	P	0.00194
108	regulation of hydrolase activity	P	0.00194
109	cytokinetic process	P	0.002064
110	peptidase inhibitor activity	F	0.002086
111	negative regulation of biosynthetic process	P	0.002089
112	regulation of protein metabolic process	P	0.002093
113	negative regulation of cellular macromolecule biosynthetic process	P	0.002208
114	cell activation	P	0.00223
115	protein binding	F	0.002239
116	regulation of endopeptidase activity	P	0.002262
117	immune system process	P	0.002347
118	regulation of biosynthetic process	P	0.002357
119	response to UV	P	0.002389
120	artery development	P	0.002408
121	cellular localization	P	0.002457
122	peptidase regulator activity	F	0.002618
123	translation	P	0.002625
124	RNA binding	F	0.002626
125	DNA-dependent ATPase activity	F	0.002637
126	response to gamma radiation	P	0.002637
127	regulation of cellular protein metabolic process	P	0.00264
128	regulation of proteolysis	P	0.00265

129	peptide metabolic process	P	0.002679
130	regulation of spindle assembly	P	0.002745
131	cerebellar cortex development	P	0.002745
132	regulation of multicellular organismal process	P	0.002783
133	retrograde transport, endosome to Golgi	P	0.002806
134	external side of plasma membrane	C	0.002806
135	negative regulation of protein processing	P	0.002851
136	negative regulation of protein maturation	P	0.002851
137	phosphatidylinositol-3,4-bisphosphate binding	F	0.002851
138	tubulin binding	F	0.00286
139	response to oxygen-containing compound	P	0.002941
140	organic acid biosynthetic process	P	0.002945
141	cellular response to chemical stimulus	P	0.002969
142	regulation of cellular component organization	P	0.003043
143	negative regulation of developmental process	P	0.003108
144	negative regulation of transcription, DNA-templated	P	0.003116
145	cellular response to oxygen radical	P	0.003121
146	protein-DNA complex disassembly	P	0.003121
147	cellular response to superoxide	P	0.003121
148	positive regulation of muscle cell differentiation	P	0.003121
149	response to superoxide	P	0.003121
150	regulation of chromosome organization	P	0.003141
151	regulation of cellular protein localization	P	0.00315
152	regulation of peptidase activity	P	0.003235
153	cytosolic transport	P	0.003263
154	regulation of phosphate metabolic process	P	0.003284
155	regulation of protein modification process	P	0.003368
156	heterocycle biosynthetic process	P	0.00341
157	regulation of cellular macromolecule biosynthetic process	P	0.003414
158	negative regulation of macromolecule biosynthetic process	P	0.003465
159	microtubule binding	F	0.003486
160	negative regulation of nucleic acid-templated transcription	P	0.003547
161	negative regulation of RNA biosynthetic process	P	0.003547
162	cellular response to ionizing radiation	P	0.003603
163	intermediate filament cytoskeleton organization	P	0.003603
164	intermediate filament-based process	P	0.003603
165	regulation of phosphorus metabolic process	P	0.003644
166	immunoglobulin production	P	0.00366
167	regulation of neurogenesis	P	0.003723
168	protein localization to chromosome	P	0.003725
169	positive regulation of binding	P	0.003725
170	endopeptidase inhibitor activity	F	0.003758
171	cell cycle process	P	0.003792
172	protein-containing complex binding	F	0.003841

173	circulatory system development	P	0.003867
174	negative regulation of cellular biosynthetic process	P	0.003946
175	immunoglobulin production involved in immunoglobulin mediated immune response	P	0.003946
176	positive regulation of DNA metabolic process	P	0.003973
177	cell differentiation	P	0.003974
178	negative regulation of cell development	P	0.004069
179	protein kinase complex	C	0.00413
180	protein kinase binding	F	0.004137
181	regulation of neuron differentiation	P	0.004166
182	blood vessel morphogenesis	P	0.004166
183	regulation of mitotic cell cycle	P	0.004236
184	carboxylic acid biosynthetic process	P	0.004305
185	regulation of cellular biosynthetic process	P	0.004332
186	serine/threonine protein kinase complex	C	0.004436
187	ribosomal subunit	C	0.004442
188	protein localization to organelle	P	0.004483
189	negative regulation of response to DNA damage stimulus	P	0.004494
190	cellular response to UV	P	0.004597
191	negative regulation of endopeptidase activity	P	0.004597
192	phosphatidylinositol bisphosphate binding	F	0.004597
193	nucleic acid metabolic process	P	0.004606
194	regulation of macromolecule biosynthetic process	P	0.004608
195	immune system development	P	0.004609
196	regulation of histone modification	P	0.004657
197	negative regulation of neurogenesis	P	0.004657
198	cellular response to organic substance	P	0.004752
199	cytokine binding	F	0.004785
200	regulation of cellular localization	P	0.005092
201	nuclear chromosome segregation	P	0.005166
202	establishment of chromosome localization	P	0.005197
203	leukocyte activation	P	0.005202
204	cellular response to reactive oxygen species	P	0.005286
205	regulation of transcription, DNA-templated	P	0.005307
206	establishment of localization	P	0.00535
207	aromatic compound biosynthetic process	P	0.005471
208	regulation of transforming growth factor beta receptor signaling pathway	P	0.005585
209	regulation of cellular response to transforming growth factor beta stimulus	P	0.005585
210	regulation of RNA biosynthetic process	P	0.005586
211	negative regulation of chromatin organization	P	0.005601
212	positive regulation of molecular function	P	0.005686
213	regulation of nucleic acid-templated transcription	P	0.005709

214	response to ammonium ion	P	0.005746
215	superoxide metabolic process	P	0.005746
216	cellular detoxification	P	0.005746
217	negative regulation of protein complex assembly	P	0.005825
218	DNA biosynthetic process	P	0.005843
219	protein stabilization	P	0.00598
220	anatomical structure formation involved in morphogenesis	P	0.006025
221	cellular response to oxygen-containing compound	P	0.006056
222	regulation of nervous system development	P	0.006137
223	cellular protein metabolic process	P	0.006182
224	reactive oxygen species metabolic process	P	0.00619
225	chromosome segregation	P	0.006244
226	negative regulation of organelle organization	P	0.006272
227	striated muscle cell differentiation	P	0.006333
228	organelle organization	P	0.006449
229	positive regulation of cellular metabolic process	P	0.006488
230	negative regulation of neuron differentiation	P	0.00663
231	negative regulation of keratinocyte proliferation	P	0.006632
232	regulation of histone H3-K9 methylation	P	0.006632
233	WW domain binding	F	0.006632
234	regulation of sterol biosynthetic process	P	0.006632
235	toll-like receptor 9 signaling pathway	P	0.006632
236	regulation of histone H3-K9 trimethylation	P	0.006632
237	regulation of cholesterol biosynthetic process	P	0.006632
238	XY body	C	0.006632
239	GAIT complex	C	0.006632
240	lymph vessel development	P	0.006632
241	protein localization	P	0.006652
242	mitotic cell cycle process	P	0.006691
243	RNA polymerase II CTD heptapeptide repeat kinase activity	F	0.006796
244	cellular response to ammonium ion	P	0.006796
245	myofibril assembly	P	0.006796
246	regulation of neuron projection development	P	0.006928
247	clathrin binding	F	0.006975
248	metencephalon development	P	0.006975
249	negative regulation of cellular response to transforming growth factor beta stimulus	P	0.006975
250	negative regulation of transforming growth factor beta receptor signaling pathway	P	0.006975
251	negative regulation of neuron projection development	P	0.007042
252	muscle cell development	P	0.007042
253	negative regulation of peptidase activity	P	0.007042
254	cellular response to light stimulus	P	0.007042
255	regulation of phosphorylation	P	0.007102

256	positive regulation of nitrogen compound metabolic process	P	0.007122
257	cytoplasmic microtubule	C	0.007271
258	chromosome localization	P	0.007461
259	positive regulation of metabolic process	P	0.007553
260	positive regulation of chromosome organization	P	0.007558
261	nucleobase-containing compound biosynthetic process	P	0.007657
262	aorta development	P	0.007839
263	cellular oxidant detoxification	P	0.007839
264	response to oxygen radical	P	0.007839
265	mitotic cell cycle	P	0.007845
266	negative regulation of programmed cell death	P	0.00822
267	regulation of organelle organization	P	0.008291
268	negative regulation of cell death	P	0.008569
269	coronary vasculature development	P	0.008638
270	icosanoid biosynthetic process	P	0.008638
271	negative regulation of intrinsic apoptotic signaling pathway in response to DNA damage	P	0.008638
272	midbody abscission	P	0.008638
273	erythrocyte development	P	0.008638
274	nucleosome disassembly	P	0.008638
275	somatic hypermutation of immunoglobulin genes	P	0.00865
276	mismatch repair complex binding	F	0.00865
277	regulation of glial cell differentiation	P	0.00865
278	negative regulation of histone methylation	P	0.00865
279	somatic diversification of immune receptors via somatic mutation	P	0.00865
280	cell surface	C	0.008671
281	somatic recombination of immunoglobulin genes involved in immune response	P	0.008672
282	isotype switching	P	0.008672
283	regulation of receptor-mediated endocytosis	P	0.008672
284	somatic diversification of immunoglobulins involved in immune response	P	0.008672
285	regulation of mitotic spindle organization	P	0.008672
286	regulation of spindle organization	P	0.008672
287	response to light stimulus	P	0.008698
288	B cell mediated immunity	P	0.008892
289	immunoglobulin mediated immune response	P	0.008892
290	organonitrogen compound biosynthetic process	P	0.008919
291	negative regulation of RNA metabolic process	P	0.008924
292	negative regulation of angiogenesis	P	0.009158
293	serine-type endopeptidase inhibitor activity	F	0.009158
294	negative regulation of cell projection organization	P	0.009396
295	cellular modified amino acid metabolic process	P	0.009484
296	negative regulation of apoptotic process	P	0.009797

297	organic cyclic compound biosynthetic process	P	0.009877
298	histone binding	F	0.010002
299	chromosome organization	P	0.010339
300	regulation of cell cycle G1/S phase transition	P	0.010459
301	mitotic spindle organization	P	0.010552
302	regulation of protein phosphorylation	P	0.010611
303	negative regulation of cell cycle G1/S phase transition	P	0.010754
304	extracellular matrix binding	F	0.010754
305	protein localization to chromatin	P	0.010754
306	detoxification	P	0.010754
307	collagen binding	F	0.010754
308	cellular response to gamma radiation	P	0.010754
309	regulation of response to DNA damage stimulus	P	0.010996
310	negative regulation of nervous system development	P	0.011173
311	transforming growth factor beta receptor signaling pathway	P	0.011173
312	hindbrain development	P	0.011245
313	negative regulation of gene expression	P	0.011295
314	cell development	P	0.011498
315	monocarboxylic acid biosynthetic process	P	0.011531
316	transcription, DNA-templated	P	0.011543
317	regulation of muscle cell differentiation	P	0.011549
318	hematopoietic or lymphoid organ development	P	0.011681
319	integral component of membrane	C	0.011823
320	cytoskeletal protein binding	F	0.011966
321	cellular macromolecule localization	P	0.012024
322	response to reactive oxygen species	P	0.012242
323	nucleic acid-templated transcription	P	0.012305
324	receptor metabolic process	P	0.012329
325	aging	P	0.012329
326	import into cell	P	0.012347
327	cellular response to stimulus	P	0.01235
328	nucleic acid binding	F	0.01243
329	regulation of binding	P	0.012568
330	plasma membrane	C	0.012834
331	RNA biosynthetic process	P	0.012837
332	positive regulation of biological process	P	0.013113
333	cellular aldehyde metabolic process	P	0.013186
334	striated muscle cell development	P	0.013186
335	amyloid-beta binding	F	0.013278
336	water-soluble vitamin metabolic process	P	0.013278
337	regulation of catalytic activity	P	0.013375
338	plasma membrane region	C	0.013527
339	apoptotic signaling pathway	P	0.01373
340	embryonic organ development	P	0.014036

341	B cell activation involved in immune response	P	0.014247
342	endocytic recycling	P	0.014247
343	muscle cell proliferation	P	0.014247
344	negative regulation of blood vessel morphogenesis	P	0.014247
345	negative regulation of vasculature development	P	0.014247
346	keratinocyte differentiation	P	0.014247
347	regulation of gene expression	P	0.014306
348	vitamin metabolic process	P	0.01458
349	cerebellum development	P	0.01458
350	regulation of localization	P	0.01481
351	cell death	P	0.014826
352	plasma membrane part	C	0.014833
353	RNA localization	P	0.014953
354	ATPase activity, coupled	F	0.015037
355	B cell activation	P	0.015097
356	lipid binding	F	0.015251
357	chromosomal part	C	0.015538
358	positive regulation of macromolecule metabolic process	P	0.015809
359	regulation of protein localization	P	0.015977
360	regulation of mitotic spindle assembly	P	0.016272
361	regulation of smooth muscle cell proliferation	P	0.016272
362	multivesicular body	C	0.016272
363	ventricular septum development	P	0.016272
364	labyrinthine layer development	P	0.016272
365	sarcomere organization	P	0.016272
366	cellular protein-containing complex localization	P	0.016272
367	intracellular non-membrane-bounded organelle	C	0.016278
368	non-membrane-bounded organelle	C	0.016278
369	negative regulation of cellular response to growth factor stimulus	P	0.016562
370	cell cycle	P	0.016755
371	cellular response to drug	P	0.017068
372	anatomical structure morphogenesis	P	0.017117
373	sensory organ morphogenesis	P	0.017209
374	regulation of cysteine-type endopeptidase activity involved in apoptotic process	P	0.017209
375	tube morphogenesis	P	0.017613
376	cellular response to stress	P	0.017647
377	immune response	P	0.017649
378	cellular protein localization	P	0.017823
379	chromatin	C	0.017826
380	epidermal cell differentiation	P	0.017993
381	axon extension	P	0.018091
382	regulation of vacuole organization	P	0.018091
383	protein kinase activator activity	F	0.018091

384	protein kinase C binding	F	0.018428
385	regulation of immunoglobulin production	P	0.018428
386	spindle assembly	P	0.01861
387	positive regulation of protein metabolic process	P	0.019126
388	cell cycle G1/S phase transition	P	0.01918
389	regulation of DNA biosynthetic process	P	0.019262
390	regulation of protein localization to cell periphery	P	0.019262
391	positive regulation of phosphorus metabolic process	P	0.019264
392	positive regulation of phosphate metabolic process	P	0.019264
393	lymphocyte activation	P	0.019276
394	positive regulation of cellular process	P	0.019423
395	positive regulation of cellular protein metabolic process	P	0.019667
396	negative regulation of organelle assembly	P	0.019671
397	pericentric heterochromatin	C	0.019671
398	peroxiredoxin activity	F	0.019671
399	solute:sodium symporter activity	F	0.019671
400	cellular response to interleukin-4	P	0.019671
401	chromatin disassembly	P	0.019671
402	positive regulation of receptor-mediated endocytosis	P	0.019671
403	regulation of lamellipodium assembly	P	0.019671
404	phosphatidylinositol phosphate kinase activity	F	0.019671
405	embryonic cranial skeleton morphogenesis	P	0.019671
406	vesicle targeting, to, from or within Golgi	P	0.019671
407	regulation of cellular response to stress	P	0.019782
408	negative regulation of cell differentiation	P	0.019926
409	heterochromatin	C	0.020378
410	negative regulation of multicellular organismal process	P	0.020389
411	hemopoiesis	P	0.020822
412	intrinsic component of membrane	C	0.020827
413	regulation of axonogenesis	P	0.020878
414	positive regulation of histone modification	P	0.020878
415	positive regulation of cellular component organization	P	0.020924
416	negative regulation of intracellular transport	P	0.021223
417	DNA damage checkpoint	P	0.021354
418	receptor-mediated endocytosis	P	0.021354
419	programmed cell death	P	0.021434
420	cell surface receptor signaling pathway	P	0.021442
421	myeloid cell differentiation	P	0.021497
422	response to stress	P	0.021661
423	cellular developmental process	P	0.021725
424	regulation of cell development	P	0.021891
425	negative regulation of DNA metabolic process	P	0.022201
426	regulation of RNA metabolic process	P	0.022205
427	cytokine receptor binding	F	0.022596

428	nuclear matrix	C	0.022596
429	negative regulation of response to stimulus	P	0.02285
430	intramolecular transferase activity	F	0.023029
431	regulation of G protein-coupled receptor signaling pathway	P	0.023029
432	regulation of extent of cell growth	P	0.023029
433	cardiac septum development	P	0.023029
434	DNA damage response, signal transduction by p53 class mediator	P	0.023029
435	fibronectin binding	F	0.023148
436	mitotic G1/S transition checkpoint	P	0.023148
437	mitotic G1 DNA damage checkpoint	P	0.023148
438	positive regulation of toll-like receptor signaling pathway	P	0.023148
439	cytokine receptor activity	F	0.023148
440	sensory perception of pain	P	0.023148
441	cardiac muscle adaptation	P	0.023148
442	protein localization to cell cortex	P	0.023148
443	response to acetylcholine	P	0.023148
444	muscle hypertrophy in response to stress	P	0.023148
445	ether metabolic process	P	0.023148
446	cellular response to acetylcholine	P	0.023148
447	regulation of cholesterol metabolic process	P	0.023148
448	response to vitamin D	P	0.023148
449	glycerol ether metabolic process	P	0.023148
450	histone H3-K9 trimethylation	P	0.023148
451	phosphorylation of RNA polymerase II C-terminal domain	P	0.023148
452	UV-damage excision repair	P	0.023148
453	cardiac muscle hypertrophy in response to stress	P	0.023148
454	positive regulation of protein localization to early endosome	P	0.023251
455	establishment of protein localization to chromatin	P	0.023251
456	glutathione biosynthetic process	P	0.023251
457	phosphotransferase activity, carboxyl group as acceptor	F	0.023251
458	post-embryonic animal organ development	P	0.023251
459	response to testosterone	P	0.023251
460	negative regulation of cholesterol metabolic process	P	0.023251
461	cobalamin binding	F	0.023251
462	CHRAC	C	0.023251
463	negative regulation of cholesterol biosynthetic process	P	0.023251
464	embryonic viscerocranium morphogenesis	P	0.023251
465	negative regulation of histone H3-K9 trimethylation	P	0.023251
466	positive regulation of ubiquitin protein ligase activity	P	0.023251
467	cell differentiation involved in embryonic placenta development	P	0.023251
468	cellular response to testosterone stimulus	P	0.023251
469	positive regulation of cell aging	P	0.023251
470	negative regulation of alcohol biosynthetic process	P	0.023251
471	negative regulation of cysteine-type endopeptidase activity involved	P	0.023251

	in apoptotic signaling pathway		
472	positive regulation of cAMP-dependent protein kinase activity	P	0.023251
473	regulation of protein localization to early endosome	P	0.023251
474	guanine/thymine mispair binding	F	0.023251
475	pituitary gland development	P	0.023251
476	phospholipase A2 activity	F	0.023251
477	thyroid gland development	P	0.023251
478	branching morphogenesis of a nerve	P	0.023251
479	Golgi to endosome transport	P	0.023251
480	citrulline biosynthetic process	P	0.023251
481	nuclear retention of pre-mRNA at the site of transcription	P	0.023251
482	MutSalpha complex binding	F	0.023251
483	pole plasm	C	0.023251
484	pyrimidine dimer repair by nucleotide-excision repair	P	0.023251
485	DNA replication factor C complex	C	0.023251
486	Leydig cell differentiation	P	0.023251
487	immunoglobulin V(D)J recombination	P	0.023251
488	lymph vessel morphogenesis	P	0.023251
489	regulation of cAMP-dependent protein kinase activity	P	0.023251
490	transferase activity, transferring aldehyde or ketonic groups	F	0.023251
491	transcription elongation from RNA polymerase I promoter	P	0.023251
492	P granule	C	0.023251
493	positive regulation of protein localization to cell cortex	P	0.023251
494	germ plasm	C	0.023251
495	negative regulation of histone H3-K9 methylation	P	0.023251
496	negative reguation of sterol biosynthetic process	P	0.023251
497	regulation of oxidative phosphorylation	P	0.02332
498	negative regulation of histone modification	P	0.02332
499	negative regulation of G1/S transition of mitotic cell cycle	P	0.02332
500	epidermis development	P	0.023552
501	endocytosis	P	0.023718
502	negative regulation of transmembrane receptor protein serine/threonine kinase signaling pathway	P	0.023971
503	growth factor binding	F	0.023971
504	vacuolar transport	P	0.024077
505	glial cell differentiation	P	0.024092
506	positive regulation of organelle organization	P	0.024162
507	phospholipid binding	F	0.024458
508	cell morphogenesis involved in neuron differentiation	P	0.024602
509	muscle structure development	P	0.024812
510	positive regulation of cell death	P	0.024828
511	regulation of organelle assembly	P	0.024872
512	apoptotic process	P	0.02518
513	DNA recombination	P	0.025342

514	positive regulation of chromatin organization	P	0.025385
515	cell cycle checkpoint	P	0.025543
516	regulation of protein localization to cell cortex	P	0.025595
517	negative regulation of autophagosome assembly	P	0.025595
518	female meiosis chromosome segregation	P	0.025595
519	polyadenylation-dependent snoRNA 3'-end processing	P	0.025595
520	DNA clamp loader activity	F	0.025595
521	thioredoxin peroxidase activity	F	0.025595
522	procollagen-proline dioxygenase activity	F	0.025595
523	cell trailing edge	C	0.025595
524	protein-DNA loading ATPase activity	F	0.025595
525	MAP kinase kinase activity	F	0.025595
526	Smc5-Smc6 complex	C	0.025595
527	positive regulation of phosphoprotein phosphatase activity	P	0.025595
528	uropod	C	0.025595
529	protein serine/threonine kinase activator activity	F	0.025595
530	SUMO ligase complex	C	0.025595
531	G protein-coupled acetylcholine receptor signaling pathway	P	0.025595
532	regulation of alcohol biosynthetic process	P	0.025595
533	MutLalpha complex binding	F	0.025595
534	intracellular signal transduction involved in G1 DNA damage checkpoint	P	0.025595
535	chemokine receptor binding	F	0.025595
536	signal transduction involved in mitotic G1 DNA damage checkpoint	P	0.025595
537	synaptic transmission, dopaminergic	P	0.025595
538	nuclear receptor binding	F	0.025595
539	DNA repair	P	0.025613
540	homeostatic process	P	0.025683
541	positive regulation of endocytosis	P	0.026354
542	negative regulation of cell proliferation	P	0.026655
543	kinase binding	F	0.026944
544	Z disc	C	0.027023
545	I band	C	0.027023
546	maintenance of protein localization in organelle	P	0.027023
547	regulation of histone methylation	P	0.027023
548	platelet-derived growth factor receptor signaling pathway	P	0.027023
549	cell proliferation	P	0.027244
550	production of molecular mediator of immune response	P	0.02744
551	cellular response to toxic substance	P	0.02744
552	regulation of immune system process	P	0.028042
553	regulation of response to stimulus	P	0.028179
554	regulation of response to stress	P	0.028346
555	cellular amino acid biosynthetic process	P	0.028402
556	mitotic cell cycle checkpoint	P	0.0289

557	positive regulation of immune response	P	0.029268
558	cellular modified amino acid biosynthetic process	P	0.029404
559	astrocyte differentiation	P	0.029404
560	tetrapyrrole metabolic process	P	0.029404
561	protein localization to chromosome, centromeric region	P	0.029404
562	negative regulation of DNA recombination	P	0.029404
563	regulation of chromatin silencing	P	0.029404
564	regulation of autophagosome assembly	P	0.029404
565	gliogenesis	P	0.029445
566	regulation of cysteine-type endopeptidase activity	P	0.029445
567	exocrine system development	P	0.029537
568	peripheral nervous system development	P	0.029537
569	smooth muscle cell proliferation	P	0.029537
570	mitotic chromosome condensation	P	0.029537
571	actin-dependent ATPase activity	F	0.029537
572	syncytium formation	P	0.029537
573	positive regulation of biosynthetic process	P	0.029543
574	regulation of biological quality	P	0.029595
575	regulation of cell death	P	0.029886
576	cellular response to biotic stimulus	P	0.030149
577	positive regulation of hydrolase activity	P	0.030179
578	regulation of vasculature development	P	0.030229
579	establishment of localization in cell	P	0.030262
580	protein-containing complex disassembly	P	0.030447
581	extrinsic apoptotic signaling pathway	P	0.030489
582	positive regulation of catalytic activity	P	0.030536
583	enzyme linked receptor protein signaling pathway	P	0.030592
584	in utero embryonic development	P	0.030633
585	negative regulation of apoptotic signaling pathway	P	0.030792
586	large ribosomal subunit	C	0.030812
587	negative regulation of hydrolase activity	P	0.030812
588	cell junction organization	P	0.030812
589	positive regulation of macromolecule biosynthetic process	P	0.030856
590	DNA-templated transcription, elongation	P	0.031628
591	RNA metabolic process	P	0.031827
592	mitotic nuclear division	P	0.03198
593	enzyme binding	F	0.032138
594	integral component of mitochondrial membrane	C	0.032869
595	carboxylic acid metabolic process	P	0.032875
596	cellular response to DNA damage stimulus	P	0.032958
597	nuclear chromosome part	C	0.033181
598	response to leukemia inhibitory factor	P	0.033319
599	plasma membrane raft	C	0.033319
600	cellular response to leukemia inhibitory factor	P	0.033319

601	ligase activity, forming carbon-nitrogen bonds	F	0.033319
602	collagen-containing extracellular matrix	C	0.033319
603	positive regulation of DNA biosynthetic process	P	0.033319
604	positive regulation of programmed cell death	P	0.033937
605	positive regulation of protein modification process	P	0.034208
606	negative regulation of signaling	P	0.034351
607	drug metabolic process	P	0.034357
608	heparin binding	F	0.034509
609	SMAD binding	F	0.034509
610	icosanoid metabolic process	P	0.034509
611	copper ion binding	F	0.034509
612	transport	P	0.034654
613	cell activation involved in immune response	P	0.034748
614	phosphatidylinositol binding	F	0.035229
615	establishment of organelle localization	P	0.035298
616	regulation of programmed cell death	P	0.03552
617	response to stimulus	P	0.035549
618	angiogenesis	P	0.035567
619	negative regulation of cell cycle	P	0.035622
620	alpha-amino acid biosynthetic process	P	0.035737
621	positive regulation of cysteine-type endopeptidase activity involved in apoptotic process	P	0.035737
622	proton transmembrane transport	P	0.035737
623	skeletal system development	P	0.035881
624	cellular response to oxidative stress	P	0.036424
625	phosphorylation	P	0.036445
626	regulation of transport	P	0.036552
627	nuclear division	P	0.036597
628	negative regulation of chromosome organization	P	0.036693
629	cell-substrate junction	C	0.036899
630	microtubule cytoskeleton organization involved in mitosis	P	0.036899
631	regulation of membrane potential	P	0.036899
632	regulation of developmental growth	P	0.036899
633	negative regulation of mitotic cell cycle	P	0.036959
634	negative regulation of cell cycle process	P	0.03702
635	cell periphery	C	0.037022
636	transferase complex, transferring phosphorus-containing groups	C	0.037309
637	hindbrain morphogenesis	P	0.03742
638	acute inflammatory response	P	0.03742
639	nuclear exosome (RNase complex)	C	0.03742
640	positive regulation of protein complex disassembly	P	0.03742
641	regulation of cytoplasmic transport	P	0.03742
642	membrane biogenesis	P	0.03742
643	response to interleukin-4	P	0.03742

644	Arp2/3 protein complex	C	0.03742
645	aminoacyl-tRNA synthetase multienzyme complex	C	0.03742
646	nucleosome positioning	P	0.03742
647	cellular response to nutrient	P	0.03742
648	phosphatidylcholine metabolic process	P	0.03742
649	mitotic cytokinetic process	P	0.03742
650	glyceraldehyde-3-phosphate metabolic process	P	0.03742
651	regulation of transcription by RNA polymerase II	P	0.037466
652	muscle adaptation	P	0.037681
653	protein localization to kinetochore	P	0.037681
654	regulation of axon extension	P	0.037681
655	membrane invagination	P	0.037681
656	maintenance of protein location in nucleus	P	0.037681
657	cyclin binding	F	0.037681
658	oxidoreductase activity, acting on the CH-NH group of donors, NAD or NADP as acceptor	F	0.037681
659	positive regulation of reactive oxygen species metabolic process	P	0.037681
660	regulation of cell cycle process	P	0.038192
661	supramolecular fiber organization	P	0.038203
662	positive regulation of cell differentiation	P	0.038332
663	synapse assembly	P	0.038733
664	protein localization to cytoskeleton	P	0.038733
665	heart development	P	0.038968
666	ATPase activity	F	0.039071
667	regulation of DNA repair	P	0.039411
668	basolateral plasma membrane	C	0.039411
669	endoplasmic reticulum lumen	C	0.039411
670	regulation of immune response	P	0.03975
671	protein folding	P	0.040039
672	protein domain specific binding	F	0.04006
673	MAPK cascade	P	0.040582
674	development of primary sexual characteristics	P	0.040769
675	organic cyclic compound binding	F	0.040943
676	cellular component disassembly	P	0.041354
677	sensory organ development	P	0.041552
678	positive regulation of phosphorylation	P	0.041594
679	negative regulation of cell communication	P	0.041818
680	intrinsic component of mitochondrial membrane	C	0.041863
681	mRNA transport	P	0.041863
682	head development	P	0.042043
683	mitotic metaphase plate congression	P	0.042197
684	positive regulation of peptidyl-serine phosphorylation	P	0.042197
685	chromosome	C	0.042532
686	leukocyte activation involved in immune response	P	0.04273

687	axonogenesis	P	0.042886
688	rRNA metabolic process	P	0.042931
689	cytosol	C	0.043247
690	skin development	P	0.043401
691	DNA integrity checkpoint	P	0.043401
692	regulation of cell cycle	P	0.043416
693	nucleobase-containing compound transport	P	0.043625
694	positive regulation of nucleobase-containing compound metabolic process	P	0.043736
695	establishment of RNA localization	P	0.043934
696	negative regulation of cell morphogenesis involved in differentiation	P	0.044228
697	digestion	P	0.044228
698	oxidoreductase activity, acting on the CH-NH group of donors	F	0.044228
699	regulation of dendrite morphogenesis	P	0.044228
700	regulation of transmembrane receptor protein serine/threonine kinase signaling pathway	P	0.044255
701	carbohydrate catabolic process	P	0.044255
702	regulation of angiogenesis	P	0.04448
703	regulation of cell morphogenesis involved in differentiation	P	0.044593
704	regulation of mitotic nuclear division	P	0.044593
705	unfolded protein binding	F	0.044609
706	monocarboxylic acid metabolic process	P	0.044775
707	cell aging	P	0.045018
708	intermediate filament	C	0.045018
709	cellular response to molecule of bacterial origin	P	0.045018
710	mesenchymal cell differentiation	P	0.045018
711	cellular response to lipopolysaccharide	P	0.045018
712	cytoskeleton organization	P	0.045454
713	protein localization to cell periphery	P	0.045541
714	supramolecular fiber	C	0.047288
715	supramolecular polymer	C	0.047288
716	supramolecular complex	C	0.047288
717	negative regulation of intrinsic apoptotic signaling pathway	P	0.047295
718	mitotic cytokinesis	P	0.047295
719	monovalent inorganic cation homeostasis	P	0.047295
720	positive regulation of response to DNA damage stimulus	P	0.047295
721	nuclear RNA surveillance	P	0.047332
722	negative regulation of extrinsic apoptotic signaling pathway via death domain receptors	P	0.047332
723	macrophage activation	P	0.047332
724	endoplasmic reticulum tubular network organization	P	0.047332
725	artery morphogenesis	P	0.047332
726	histone H3-K9 methylation	P	0.047332
727	response to vitamin	P	0.047332

728	regulation of oxidative stress-induced intrinsic apoptotic signaling pathway	P	0.047332
729	positive regulation of vacuole organization	P	0.047332
730	respiratory burst	P	0.047332
731	microtubule plus-end binding	F	0.047332
732	actin filament-based transport	P	0.047332
733	G1 DNA damage checkpoint	P	0.047332
734	regulation of early endosome to late endosome transport	P	0.047332
735	negative regulation of protein acetylation	P	0.047332
736	striated muscle adaptation	P	0.047332
737	glutamine family amino acid biosynthetic process	P	0.047332
738	ESC/E(Z) complex	C	0.047332
739	cerebellar Purkinje cell layer development	P	0.047332
740	aldehyde catabolic process	P	0.047332
741	nuclear mRNA surveillance	P	0.047332
742	positive regulation of epithelial to mesenchymal transition	P	0.047332
743	negative regulation of transcription by RNA polymerase II	P	0.048177
744	side of membrane	C	0.048263
745	bone mineralization	P	0.048506
746	regulation of immunoglobulin mediated immune response	P	0.048506
747	cytochrome complex	C	0.048506
748	positive regulation of protein dephosphorylation	P	0.048506
749	intrinsic apoptotic signaling pathway in response to DNA damage by p53 class mediator	P	0.048506
750	regulation of isotype switching	P	0.048506
751	microtubule end	C	0.048506
752	regulation of B cell mediated immunity	P	0.048506
753	mRNA cleavage	P	0.048506
754	cardiac chamber development	P	0.049116
755	lymphocyte activation involved in immune response	P	0.049116
756	cardiocyte differentiation	P	0.049435
757	cargo receptor activity	F	0.049435
758	receptor internalization	P	0.049435
759	chromosome condensation	P	0.049435
760	retina development in camera-type eye	P	0.049435
761	protein localization to microtubule cytoskeleton	P	0.049435
762	'de novo' protein folding	P	0.049435
763	chordate embryonic development	P	0.049435
764	negative regulation of protein metabolic process	P	0.049697
