# Supplementary material for: Proteomics Reveals the Response Mechanism of Embryonic Bovine Lung Cells to Mycoplasma bovis Infection
Source: Int J Mol Sci. 2025 Jan 19;26(2):823. doi: 10.3390/ijms26020823 (PMC11765741; doi:10.3390/ijms26020823)
Supplement: Supplementary file 1 [file ijms-26-00823-s001.zip › KEGG.pdf]

Number	secondary classification	12 hpi Map Name	P value
1	Immune system	Neutrophil extracellular trap formation	0.003303229
2	Amino acid metabolism	Cysteine and methionine metabolism	0.004624366
3	Immune system	Chemokine signaling pathway	0.012485929
4	Carbohydrate metabolism	Glycolysis / Gluconeogenesis	0.018817236
5	Cellular community - eukaryotes	Gap junction	0.018817236
6		Biosynthesis of nucleotide sugars	0.031533904
7	Immune system	IL-17 signaling pathway	0.031533904
8	Nervous system	Serotonergic synapse	0.031533904
9	Signal transduction	PI3K-Akt signaling pathway	0.034964815
10	Endocrine system	Thyroid hormone signaling pathway	0.035880408
11	Carbohydrate metabolism	Propanoate metabolism	0.041571799
12	Carbohydrate metabolism	Pentose phosphate pathway	0.050716072
13	Immune system	Complement and coagulation cascades	0.050716072
14	Carbohydrate metabolism	Amino sugar and nucleotide sugar metabolism	0.057561068
15	Signaling molecules and interaction	Cell adhesion molecules	0.060501633
16	Endocrine system	Glucagon signaling pathway	0.063681503
17	Endocrine system	Relaxin signaling pathway	0.063681503
18	Signal transduction	TGF-beta signaling pathway	0.070089193
19	Endocrine system	GnRH secretion	0.070868682
20	Digestive system	Protein digestion and absorption	0.070868682
21	Immune system	NOD-like receptor signaling pathway	0.079100772
22	Signal transduction	Ras signaling pathway	0.084411739
23	Endocrine system	Progesterone-mediated oocyte maturation	0.090944235
24	Signaling molecules and interaction	Cytokine-cytokine receptor interaction	0.093125501
25	Cell growth and death	Apoptosis	0.09554044
26	Folding, sorting and degradation	SNARE interactions in vesicular transport	0.1049126
27	Immune system	Th17 cell differentiation	0.1049126
28	Metabolism of other amino acids	Taurine and hypotaurine metabolism	0.107260702
29	Metabolism of cofactors and vitamins	Vitamin B6 metabolism	0.107260702
30	Signaling molecules and interaction	Viral protein interaction with cytokine and cytokine receptor	0.107260702
31	Sensory system	Phototransduction	0.107260702
32	Cellular community - eukaryotes	Tight junction	0.113446702
33		Nucleotide metabolism	0.114037765
34	Nervous system	Long-term depression	0.117075516
35	Signal transduction	HIF-1 signaling pathway	0.122183807
36	Endocrine system	Oxytocin signaling pathway	0.122183807

37	Endocrine system	Estrogen signaling pathway	0.130535749
38	Signal transduction	Apelin signaling pathway	0.139082532
39	Sensory system	Taste transduction	0.140402984
40	Circulatory system	Vascular smooth muscle contraction	0.142356051
41	Environmental adaptation	Circadian entrainment	0.142356051
42	Digestive system	Pancreatic secretion	0.142356051
43	Carbohydrate metabolism	Pyruvate metabolism	0.155393951
44	Immune system	B cell receptor signaling pathway	0.155393951
45	Nervous system	Long-term potentiation	0.155393951
46	Endocrine system	GnRH signaling pathway	0.155393951
47	Endocrine system	Parathyroid hormone synthesis, secretion and action	0.155393951
48	Cell growth and death	Cell cycle	0.160302714
49	Immune system	Natural killer cell mediated cytotoxicity	0.168647952
50	Metabolism of cofactors and vitamins	Pantothenate and CoA biosynthesis	0.172319304
51	Nucleotide metabolism	Purine metabolism	0.174996536
52	Immune system	Toll-like receptor signaling pathway	0.182084242
53	Nervous system	Glutamatergic synapse	0.182084242
54	Nervous system	Dopaminergic synapse	0.182084242
55	Endocrine system	Melanogenesis	0.182084242
56	Immune system	C-type lectin receptor signaling pathway	0.195671203
57	Nervous system	Cholinergic synapse	0.195671203
58	Signal transduction	TNF signaling pathway	0.223180954
59	Digestive system	Carbohydrate digestion and absorption	0.232653145
60	Transport and catabolism	Phagosome	0.23285398
61	Nervous system	Retrograde endocannabinoid signaling	0.23285398
62	Cellular community - eukaryotes	Signaling pathways regulating pluripotency of stem cells	0.237050471
63	Endocrine system	Growth hormone synthesis, secretion and action	0.237050471
64	Signal transduction	Phospholipase D signaling pathway	0.250963911
65	Signal transduction	Hippo signaling pathway	0.250963911
66	Metabolism of other amino acids	beta-Alanine metabolism	0.261156119
67	Glycan biosynthesis and metabolism	Mucin type O-glycan biosynthesis	0.261156119
68	Metabolism of cofactors and vitamins	Lipoic acid metabolism	0.261156119
69	Signal transduction	cGMP-PKG signaling pathway	0.278835069
70	Signal transduction	Sphingolipid signaling pathway	0.278835069
71	Carbohydrate metabolism	Starch and sucrose metabolism	0.288604159
72	Signaling molecules and interaction	ECM-receptor interaction	0.288604159
73	Endocrine system	Insulin secretion	0.288604159

74	Transport and catabolism	Autophagy - animal	0.314052391
75	Metabolism of cofactors and vitamins	Nicotinate and nicotinamide metabolism	0.315036168
76	Immune system	T cell receptor signaling pathway	0.320464134
77		Motor proteins	0.324325166
78	Amino acid metabolism	Glycine, serine and threonine metabolism	0.340489622
79	Excretory system	Endocrine and other factor-regulated calcium reabsorption	0.340489622
80	Signal transduction	AMPK signaling pathway	0.347906026
81	Endocrine system	Renin secretion	0.365000616
82	Endocrine system	Cortisol synthesis and secretion	0.365000616
83	Digestive system	Salivary secretion	0.365000616
84	Glycan biosynthesis and metabolism	Other types of O-glycan biosynthesis	0.388603922
85	Signal transduction	Notch signaling pathway	0.388603922
86	Signal transduction	VEGF signaling pathway	0.388603922
87	Immune system	Th1 and Th2 cell differentiation	0.388603922
88	Immune system	Fc epsilon RI signaling pathway	0.388603922
89	Excretory system	Vasopressin-regulated water reabsorption	0.388603922
90	Transport and catabolism	Endocytosis	0.396552829
91	Sensory system	Inflammatory mediator regulation of TRP channels	0.411333031
92	Digestive system	Mineral absorption	0.411333031
93	Development and regeneration	Axon guidance	0.4146678
94	Energy metabolism	Oxidative phosphorylation	0.416001535
95	Nervous system	GABAergic synapse	0.433220205
96	Digestive system	Gastric acid secretion	0.433220205
97	Signal transduction	Rap1 signaling pathway	0.440426752
98	Cell growth and death	Oocyte meiosis	0.453072431
99	Cell growth and death	Necroptosis	0.465554116
100	Carbohydrate metabolism	Inositol phosphate metabolism	0.474591901
101	Lipid metabolism	Glycerophospholipid metabolism	0.474591901
102	Nervous system	Synaptic vesicle cycle	0.474591901
103	Endocrine system	Thyroid hormone synthesis	0.474591901
104	Cell growth and death	Ferroptosis	0.494135186
105	Immune system	RIG-I-like receptor signaling pathway	0.494135186
106	Signal transduction	JAK-STAT signaling pathway	0.494135186
107	Endocrine system	Prolactin signaling pathway	0.494135186
108	Endocrine system	Aldosterone synthesis and secretion	0.494135186
109	Glycan biosynthesis and metabolism	Various types of N-glycan biosynthesis	0.512954144
110	Xenobiotics biodegradation and metabolism	Drug metabolism - other enzymes	0.512954144

111	Development and regeneration	Osteoclast differentiation	0.512954144
112	Immune system	Antigen processing and presentation	0.512954144
113	Endocrine system	Adipocytokine signaling pathway	0.512954144
114	Translation	Ribosome	0.521905929
115	Cellular community - eukaryotes	Focal adhesion	0.525338955
116	Amino acid metabolism	Valine, leucine and isoleucine degradation	0.531075522
117		Viral life cycle - HIV-1	0.531075522
118	Signal transduction	NF-kappa B signaling pathway	0.531075522
119	Signal transduction	Phosphatidylinositol signaling system	0.531075522
120	Glycan biosynthesis and metabolism	N-Glycan biosynthesis	0.548525086
121	Signal transduction	MAPK signaling pathway	0.55899131
122		Polycomb repressive complex	0.565327652
123	Endocrine system	PPAR signaling pathway	0.581507123
124	Signal transduction	Calcium signaling pathway	0.581507123
125	Signal transduction	ErbB signaling pathway	0.597086522
126	Immune system	Leukocyte transendothelial migration	0.597086522
127	Aging	Longevity regulating pathway - multiple species	0.612088024
128	Signal transduction	Wnt signaling pathway	0.626532988
129	Immune system	Cytosolic DNA-sensing pathway	0.626532988
130	Immune system	Platelet activation	0.640441988
131	Immune system	Fc gamma R-mediated phagocytosis	0.640441988
132	Folding, sorting and degradation	Proteasome	0.653834836
133	Nervous system	Neurotrophin signaling pathway	0.653834836
134	Environmental adaptation	Thermogenesis	0.655153089
135	Circulatory system	Adrenergic signaling in cardiomyocytes	0.66673062
136		Alcoholic liver disease	0.66673062
137	Signal transduction	FoxO signaling pathway	0.713700718
138	Signal transduction	cAMP signaling pathway	0.734650269
139	Endocrine system	Insulin signaling pathway	0.734650269
140	Transport and catabolism	Lysosome	0.754072168
141	Signal transduction	mTOR signaling pathway	0.763245259
142	Cell growth and death	Cellular senescence	0.788769008
143	Transcription	Spliceosome	0.803419074
144		ATP-dependent chromatin remodeling	0.804242435
145	Folding, sorting and degradation	Ubiquitin mediated proteolysis	0.804242435
146	Translation	mRNA surveillance pathway	0.811550281
147	Global and overview maps	Biosynthesis of cofactors	0.84420806
148	Cell motility	Regulation of actin cytoskeleton	0.893569118
149	Translation	Nucleocytoplasmic transport	0.901384781
150	Folding, sorting and degradation	Protein processing in endoplasmic reticulum	0.948475826

Number	secondary classification	24 hpi MapName	P value
1	Translation	Ribosome	3.17284E-07
2	Carbohydrate metabolism	Glycolysis / Gluconeogenesis	0.004597115
3	Immune system	Neutrophil extracellular trap formation	0.004597115
4	Signaling molecules and interaction	Cytokine-cytokine receptor interaction	0.005746234
5	Carbohydrate metabolism	Pentose phosphate pathway	0.007838597
6		ATP-dependent chromatin remodeling	0.009127698
7	Signal transduction	cGMP-PKG signaling pathway	0.013186015
8	Circulatory system	Vascular smooth muscle contraction	0.014579523
9	Endocrine system	Estrogen signaling pathway	0.016561789
10	Endocrine system	Progesterone-mediated oocyte maturation	0.018920227
11	Signal transduction	cAMP signaling pathway	0.022595874
12	Signaling molecules and interaction	Viral protein interaction with cytokine and cytokine receptor	0.023251011
13	Immune system	Fc gamma R-mediated phagocytosis	0.023971394
14	Signal transduction	HIF-1 signaling pathway	0.028402461
15	Endocrine system	Oxytocin signaling pathway	0.028402461
16	Endocrine system	Renin secretion	0.029537082
17	Endocrine system	Glucagon signaling pathway	0.030434334
18	Circulatory system	Cardiac muscle contraction	0.034509276
19	Immune system	Complement and coagulation cascades	0.037419763
20	Signal transduction	VEGF signaling pathway	0.048506159
21	Endocrine system	Melanogenesis	0.049435422
22	Cell growth and death	Oocyte meiosis	0.051664865
23	Environmental adaptation	Thermogenesis	0.057464274
24		Efferocytosis	0.059289358
25	Signaling molecules and interaction	Neuroactive ligand-receptor interaction	0.059484261
26	Lipid metabolism	Fatty acid degradation	0.063308086
27	Immune system	Antigen processing and presentation	0.063308086
28	Nervous system	Long-term potentiation	0.063308086
29	Signal transduction	Rap1 signaling pathway	0.079017532
30	Carbohydrate metabolism	Starch and sucrose metabolism	0.081907997
31	Endocrine system	Insulin secretion	0.081907997
32	Cell motility	Regulation of actin cytoskeleton	0.091153514
33	Replication and repair	Fanconi anemia pathway	0.096058016
34	Endocrine system	GnRH secretion	0.096058016
35	Transport and catabolism	Peroxisome	0.096658984
36	Signal transduction	PI3K-Akt signaling pathway	0.102951524
37	Energy metabolism	Oxidative phosphorylation	0.104794712

38	Immune system	Th17 cell differentiation	0.105201958
39	Amino acid metabolism	Arginine and proline metabolism	0.11114646
40	Endocrine system	Aldosterone synthesis and secretion	0.11114646
41	Cell growth and death	Necroptosis	0.116263172
42	Immune system	Fc epsilon RI signaling pathway	0.136816751
43	Endocrine system	Thyroid hormone signaling pathway	0.144354883
44	Development and regeneration	Osteoclast differentiation	0.145938006
45	Immune system	B cell receptor signaling pathway	0.145938006
46	Endocrine system	Parathyroid hormone synthesis, secretion and action	0.145938006
47	Cellular community - eukaryotes	Gap junction	0.14705451
48	Nervous system	Neurotrophin signaling pathway	0.14705451
49	Cellular community - eukaryotes	Tight junction	0.155460797
50	Endocrine system	Regulation of lipolysis in adipocytes	0.168532991
51	Signal transduction	mTOR signaling pathway	0.16918811
52	Development and regeneration	Axon guidance	0.16918811
53	Transport and catabolism	Endocytosis	0.17114825
54		Motor proteins	0.174822345
55	Signal transduction	Calcium signaling pathway	0.183794522
56	Amino acid metabolism	Alanine, aspartate and glutamate metabolism	0.184160122
57	Immune system	Natural killer cell mediated cytotoxicity	0.185316178
58	Metabolism of other amino acids	Taurine and hypotaurine metabolism	0.197972203
59	Signal transduction	FoxO signaling pathway	0.208298865
60	Nervous system	Glutamatergic synapse	0.228620138
61	Lipid metabolism	Ether lipid metabolism	0.22927648
62	Immune system	Hematopoietic cell lineage	0.22927648
63	Cell growth and death	Ferroptosis	0.233111566
64	Digestive system	Pancreatic secretion	0.233111566
65	Nervous system	GABAergic synapse	0.236798897
66	Digestive system	Gastric acid secretion	0.236798897
67	Carbohydrate metabolism	Propanoate metabolism	0.237832986
68	Replication and repair	Mismatch repair	0.237832986
69	Endocrine system	Cortisol synthesis and secretion	0.238921641
70	Digestive system	Protein digestion and absorption	0.238921641
71	Cell growth and death	Cell cycle	0.252476696
72	Immune system	NOD-like receptor signaling pathway	0.254036
73	Signal transduction	Sphingolipid signaling pathway	0.25837729
74	Cell growth and death	Cellular senescence	0.262214113
75	Translation	Aminoacyl-tRNA biosynthesis	0.266520332
76	Endocrine system	Growth hormone synthesis, secretion and action	0.266520332
77	Immune system	Chemokine signaling pathway	0.270860302

78	Nervous system	Cholinergic synapse	0.275048165
79	Endocrine system	Insulin signaling pathway	0.280032311
80	Endocrine system	GnRH signaling pathway	0.28396281
81	Biosynthesis of other secondary metabolites	Neomycin, kanamycin and gentamicin biosynthesis	0.285516477
82	Transport and catabolism	Phagosome	0.286813242
83	Nervous system	Long-term depression	0.293232018
84	Circulatory system	Adrenergic signaling in cardiomyocytes	0.300255883
85	Replication and repair	Base excision repair	0.302737198
86	Excretory system	Vasopressin-regulated water reabsorption	0.302737198
87	Nucleotide metabolism	Purine metabolism	0.307197072
88	Signal transduction	Phospholipase D signaling pathway	0.311510788
89	Signal transduction	Wnt signaling pathway	0.311510788
90	Metabolism of cofactors and vitamins	One carbon pool by folate	0.312124156
91	Metabolism of cofactors and vitamins	Nicotinate and nicotinamide metabolism	0.312124156
92	Excretory system	Collecting duct acid secretion	0.312124156
93	Folding, sorting and degradation	RNA degradation	0.318448482
94	Amino acid metabolism	Tyrosine metabolism	0.320284498
95	Excretory system	Aldosterone-regulated sodium reabsorption	0.320284498
96	Digestive system	Carbohydrate digestion and absorption	0.320284498
97	Lipid metabolism	Steroid hormone biosynthesis	0.32283529
98	Glycan biosynthesis and metabolism	Glycosaminoglycan biosynthesis - chondroitin sulfate / dermatan sulfate	0.32283529
99	Endocrine system	Renin-angiotensin system	0.32283529
100	Sensory system	Olfactory transduction	0.32283529
101	Sensory system	Taste transduction	0.32283529
102	Endocrine system	PPAR signaling pathway	0.323716775
103	Signal transduction	Phosphatidylinositol signaling system	0.337080227
104	Carbohydrate metabolism	Inositol phosphate metabolism	0.351896378
105	Transcription	RNA polymerase	0.351896378
106	Endocrine system	Thyroid hormone synthesis	0.351896378
107	Immune system	Platelet activation	0.357956605
108	Sensory system	Inflammatory mediator regulation of TRP channels	0.368613352
109	Digestive system	Mineral absorption	0.368613352
110	Amino acid metabolism	Glycine, serine and threonine metabolism	0.387969314
111	Excretory system	Endocrine and other factor-regulated calcium reabsorption	0.387969314

112	Digestive system	Bile secretion	0.387969314
113	Glycan biosynthesis and metabolism	N-Glycan biosynthesis	0.391310271
114	Replication and repair	Nucleotide excision repair	0.405125754
115	Nervous system	Retrograde endocannabinoid signaling	0.405995236
116	Cell growth and death	p53 signaling pathway	0.411288783
117	Signal transduction	JAK-STAT signaling pathway	0.411288783
118	Environmental adaptation	Circadian entrainment	0.411288783
119	Metabolism of other amino acids	beta-Alanine metabolism	0.411335402
120	Aging	Longevity regulating pathway - multiple species	0.424162158
121	Cellular community - eukaryotes	Signaling pathways regulating pluripotency of stem cells	0.424162158
122	Endocrine system	Relaxin signaling pathway	0.424162158
123	Transcription	Spliceosome	0.428262181
124		Nucleotide metabolism	0.432589035
125	Lipid metabolism	Biosynthesis of unsaturated fatty acids	0.434520579
126	Lipid metabolism	Fatty acid biosynthesis	0.441805236
127	Amino acid metabolism	Histidine metabolism	0.441805236
128	Amino acid metabolism	Phenylalanine metabolism	0.441805236
129	Metabolism of cofactors and vitamins	Pantothenate and CoA biosynthesis	0.441805236
130	Energy metabolism	Sulfur metabolism	0.441805236
131	Xenobiotics biodegradation and metabolism	Drug metabolism - cytochrome P450	0.441805236
132		Polycomb repressive complex	0.445565921
133	Carbohydrate metabolism	Galactose metabolism	0.46249315
134	Lipid metabolism	Fatty acid elongation	0.46249315
135	Lipid metabolism	Sphingolipid metabolism	0.46249315
136	Digestive system	Salivary secretion	0.46249315
137	Folding, sorting and degradation	Protein processing in endoplasmic reticulum	0.469901201
138	Amino acid metabolism	Cysteine and methionine metabolism	0.470054617
139	Glycan biosynthesis and metabolism	Various types of N-glycan biosynthesis	0.470054617
140	Xenobiotics biodegradation and metabolism	Drug metabolism - other enzymes	0.470054617
141		Biosynthesis of nucleotide sugars	0.470054617
142	Endocrine system	Adipocytokine signaling pathway	0.470054617
143	Signal transduction	AMPK signaling pathway	0.476952833
144	Cellular community - eukaryotes	Focal adhesion	0.480376578
145		Virion - Herpesvirus	0.489541546
146	Metabolism of cofactors and vitamins	Retinol metabolism	0.49808661
147	Signaling molecules and interaction	ECM-receptor interaction	0.49808661

148		Viral life cycle - HIV-1	0.527043642
149	Carbohydrate metabolism	Glyoxylate and dicarboxylate metabolism	0.533495795
150	Immune system	Th1 and Th2 cell differentiation	0.533495795
151	Xenobiotics biodegradation and metabolism	Metabolism of xenobiotics by cytochrome P450	0.548069614
152	Signal transduction	ErbB signaling pathway	0.550410631
153	Signal transduction	TNF signaling pathway	0.550410631
154	Immune system	Leukocyte transendothelial migration	0.550410631
155	Replication and repair	DNA replication	0.559928601
156	Nervous system	Synaptic vesicle cycle	0.559928601
157	Signal transduction	Apelin signaling pathway	0.5628987
158	Immune system	Toll-like receptor signaling pathway	0.58133699
159	Lipid metabolism	Primary bile acid biosynthesis	0.635326218
160	Glycan biosynthesis and metabolism	Glycosphingolipid biosynthesis - lacto and neolacto series	0.635326218
161	Metabolism of cofactors and vitamins	Vitamin B6 metabolism	0.635326218
162	Immune system	Intestinal immune network for IgA production	0.635326218
163	Sensory system	Phototransduction	0.635326218
164	Signal transduction	MAPK signaling pathway	0.587909955
165	Nucleotide metabolism	Pyrimidine metabolism	0.617097762
166	Metabolism of other amino acids	Glutathione metabolism	0.617097762
167	Transcription	Basal transcription factors	0.617097762
168	Immune system	RIG-I-like receptor signaling pathway	0.617097762
169	Endocrine system	Prolactin signaling pathway	0.617097762
170	Signal transduction	TGF-beta signaling pathway	0.645662248
171	Signal transduction	Hippo signaling pathway	0.645662248
172	Lipid metabolism	Glycerolipid metabolism	0.648965614
173	Digestive system	Cholesterol metabolism	0.659422661
174	Aging	Longevity regulating pathway	0.66961317
175	Immune system	IL-17 signaling pathway	0.66961317
176	Nervous system	Serotonergic synapse	0.66961317
177	Immune system	T cell receptor signaling pathway	0.697247027
178	Metabolism of cofactors and vitamins	Porphyrin metabolism	0.711123102
179	Glycan biosynthesis and metabolism	Mucin type O-glycan biosynthesis	0.71513308
180	Endocrine system	Ovarian steroidogenesis	0.71513308
181	Carbohydrate metabolism	Amino sugar and nucleotide sugar metabolism	0.72229659
182	Folding, sorting and degradation	Proteasome	0.727901493
183	Metabolism of cofactors and vitamins	Ubiquinone and other terpenoid-quinone biosynthesis	0.739489861
184	Glycan biosynthesis and metabolism	Mannose type O-glycan biosynthesis	0.739489861
185	Lipid metabolism	alpha-Linolenic acid metabolism	0.739489861

186	Metabolism of cofactors and vitamins	Thiamine metabolism	0.739489861
187	Translation	mRNA surveillance pathway	0.750871544
188	Lipid metabolism	Glycerophospholipid metabolism	0.760076221
189	Amino acid metabolism	Lysine degradation	0.764411294
190	Lipid metabolism	Steroid biosynthesis	0.777146357
191	Amino acid metabolism	Arginine biosynthesis	0.777146357
192	Signal transduction	Hedgehog signaling pathway	0.777146357
193	Environmental adaptation	Circadian rhythm	0.777146357
194	Global and overview maps	Biosynthesis of cofactors	0.782705017
195	Transport and catabolism	Autophagy - animal	0.786813878
196	Folding, sorting and degradation	Protein export	0.797021441
197	Cellular community - eukaryotes	Adherens junction	0.797021441
198	Immune system	C-type lectin receptor signaling pathway	0.797021441
199	Glycan biosynthesis and metabolism	Glycosaminoglycan biosynthesis - heparan sulfate / heparin	0.813911018
200	Glycan biosynthesis and metabolism	Glycosphingolipid biosynthesis - globo and isoglobo series	0.813911018
201	Energy metabolism	Nitrogen metabolism	0.813911018
202	Replication and repair	Non-homologous end-joining	0.813911018
203	Folding, sorting and degradation	Sulfur relay system	0.813911018
204	Digestive system	Vitamin digestion and absorption	0.813911018
205	Transport and catabolism	Autophagy - other	0.826986745
206	Carbohydrate metabolism	Pyruvate metabolism	0.835944287
207	Folding, sorting and degradation	SNARE interactions in vesicular transport	0.846895676
208	Amino acid metabolism	Valine, leucine and isoleucine degradation	0.865646272
209	Metabolism of other amino acids	Selenocompound metabolism	0.867079298
210	Excretory system	Proximal tubule bicarbonate reclamation	0.867079298
211	Signal transduction	Ras signaling pathway	0.867700041
212		Alcoholic liver disease	0.876490268
213	Nervous system	Dopaminergic synapse	0.890604537
214	Cell growth and death	Apoptosis	0.901059429
215	Immune system	Cytosolic DNA-sensing pathway	0.902809399
216	Carbohydrate metabolism	Pentose and glucuronate interconversions	0.905061905
217	Glycan biosynthesis and metabolism	Other glycan degradation	0.905061905
218	Metabolism of cofactors and vitamins	Folate biosynthesis	0.905061905
219	Glycan biosynthesis and metabolism	Other types of O-glycan biosynthesis	0.92185368
220	Signal transduction	Notch signaling pathway	0.92185368
221	Digestive system	Fat digestion and absorption	0.932194594
222	Translation	Ribosome biogenesis in eukaryotes	0.94695547

223	Transport and catabolism	Mitophagy - animal	0.94695547
224	Carbohydrate metabolism	Butanoate metabolism	0.951575623
225	Signal transduction	NF-kappa B signaling pathway	0.952945653
226	Translation	Nucleocytoplasmic transport	0.958284362
227	Amino acid metabolism	Tryptophan metabolism	0.965418832
228	Replication and repair	Homologous recombination	0.965418832
229	Carbohydrate metabolism	Fructose and mannose metabolism	0.975306016
230	Cell growth and death	Apoptosis - multiple species	0.975306016
231	Signaling molecules and interaction	Cell adhesion molecules	0.975306016
232	Metabolism of terpenoids and polyketides	Terpenoid backbone biosynthesis	0.987410113
233	Transport and catabolism	Lysosome	0.991016496
234	Folding, sorting and degradation	Ubiquitin mediated proteolysis	0.997891352
